# Health-related quality of life impact of minor and major bleeding events during dual antiplatelet therapy: a systematic literature review and patient preference elicitation study

**DOI:** 10.1186/s12955-018-1019-3

**Published:** 2018-09-20

**Authors:** Brett Doble, Maria Pufulete, Jessica M. Harris, Tom Johnson, Daniel Lasserson, Barnaby C. Reeves, Sarah Wordsworth

**Affiliations:** 1 Health Economics Research Centre, Nuffield Department of Population Health, University of Oxford, Oxford, OX3 7LF UK; 2 Clinical Trials and Evaluation Unit, University of Bristol, Bristol, BS2 8HW UK; 3 Bristol Heart Institute, University Hospitals Bristol National Health Service Foundation Trust, Bristol, BS2 8HJ UK; 4 Nuffield Department of Medicine, University of Oxford, Oxford, OX3 9DU UK; 5 Institute of Applied Health Research, College of Medical and Dental Sciences, University of Birmingham, B15 2TT, Birmingham, UK

**Keywords:** Aspirin, Clopidogrel, EQ-5D, Health state utility values, Prasugrel, Ticagrelor, Utility decrements

## Abstract

**Background:**

Dual antiplatelet therapy (DAPT) is the recommended preventative treatment for secondary ischaemic events, but increases the risk of bleeding, potentially affecting patients’ health-related quality-of-life (HRQoL). Varied utility decrements have been used in cost-effectiveness models assessing alternative DAPT regimens, but it is unclear which of these decrements are most appropriate. Therefore, we reviewed existing sources of utility decrements for bleeds in patients receiving DAPT and undertook primary research to estimate utility decrements through a patient elicitation exercise using vignettes and the EuroQol EQ-5D.

**Methods:**

MEDLINE, PubMed and references of included studies were searched. Primary research and decision analytic modelling studies reporting utility decrements for bleeds related to DAPT were considered. For the primary research study, 21 participants completed an elicitation exercise involving vignettes describing minor and major bleeds and the EQ-5D-3 L and EQ-5D-5 L. Utility decrements were derived using linear regression and compared to existing estimates.

**Results:**

Four hundred forty-two citations were screened, of which 12 studies were included for review. Reported utility decrements ranged from − 0.002 to − 0.03 for minor bleeds and − 0.007 to − 0.05 for major bleeds. Data sources used to estimate the decrements, however, lacked relevance to our population group and few studies adequately reported details of their measurement and valuation approaches. No study completely adhered to reimbursement agency requirements in the UK according to the National Institute for Health and Care Excellence reference case. Our primary research elicited utility decrements overlapped existing estimates, ranging from − 0.000848 to − 0.00828 for minor bleeds and − 0.0187 to − 0.0621 for major bleeds. However, the magnitude of difference depended on the instrument, estimation method and valuation approach applied.

**Conclusions:**

Several sources of utility decrements for bleeds are available for use in cost-effectiveness analyses, but are of limited quality and relevance. Our elicitation exercise has derived utility decrements from a relevant patient population, based on standardised definitions of minor and major bleeding events, using a validated HRQoL instrument and have been valued using general population tariffs. We suggest that our utility decrements be used in future cost-effectiveness analyses of DAPT.

**Electronic supplementary material:**

The online version of this article (10.1186/s12955-018-1019-3) contains supplementary material, which is available to authorized users.

## Background

Dual antiplatelet therapy (DAPT), which is a combination of aspirin and a P2Y_12_ inhibitor (clopidogrel, prasugrel or ticagrelor), with a suggested treatment duration of 6–36 months, [1] is the recommended preventative treatment for secondary ischaemic events after coronary interventions such as, percutaneous coronary intervention (PCI), coronary artery bypass grafting (CABG) and medical management of acute coronary syndrome (ACS) [2]. Recently developed P2Y_12_ inhibitors (prasugrel and ticagrelor) are associated with more potent antiplatelet efficacy [3, 4] and reduced rates of non-responsiveness [5] compared to clopidogrel, but come with increased bleeding complications and costs (approximately 25 times the cost of clopidogrel) [6]. A decision regarding balancing the risk of bleeds and costs with the expected benefit in ischaemic event reduction must, therefore, be made.

Population rates of bleeds when taking DAPT for the newer P2Y_12_ inhibitors are relatively unknown, but initial registry data suggests approximately 14% of patients on ticagrelor experience a bleed [7]. Population rates are predicted to be higher than reported in clinical trials due to the exclusion of high-risk patients and because only events requiring hospitalisation are generally reported in trials. The incidence of minor bleeds, which might not result in patients seeking medical care, has therefore not been well quantified with estimates ranging from 9 to 38% of patients on DAPT being affected [8–12]. Despite being considered minor, these events can impact on patients’ adherence to treatment, [13] thereby increasing their risk of secondary ischaemic events.

A lack of reliable estimates on the health-related quality-of-life (HRQoL) impacts of bleeds could lead to inappropriate decisions about which DAPT regimens to use in clinical practice. It is not clear to what extent primary research has determined the impact of bleeding events on HRQoL or what evidence has been used to populate existing decision analytic models assessing DAPT. Furthermore, the National Institute for Health and Care Excellence (NICE) in the United Kingdom (UK) requires the use of the EQ-5D-3 L, a generic health status questionnaire, [14] when assessing the HRQoL impacts of interventions [15]. It is, therefore, important to identify whether or not health-state utility decrements for bleeding events (hereafter referred to as ‘utility decrements’) derived from the EQ-5D-3 L are available for use in cost-effectiveness analyses. The EQ-5D-3 L has been shown to be a valid, reliable and responsive instrument to measure HRQoL in patients with ACS, [16, 17] and is a suitable questionnaire to use to derive such utility decrements. However, it is unclear if the recently developed EQ-5D-5 L, with improved sensitivity and reduced ceiling effects, [18] would also be a suitable instrument to estimate the impact of bleeding on HRQoL.

Therefore, our study first aimed to review the evidence regarding utility decrements of bleeding events in patients receiving DAPT after coronary interventions. Second, we sought to derive robust UK utility decrements for use in future cost-effectiveness analyses of DAPT, through a patient elicitation exercise using vignettes and both the 3 and 5 level versions of the EQ-5D.

## Methods

### Literature review and quality assessment

The Preferred Reporting Items for Systematic Reviews and Meta-Analyses (PRSIMA) statement [19] was used as a guideline for the design of the review, with adaptations made due to the focus of utility decrements.

#### Eligibility criteria

Studies published in English, which reported utility decrements associated with bleeds in adults taking DAPT were considered. Included studies could be primary research that prospectively collected HRQoL information from which utility decrements could be estimated or decision analytic models of DAPT that incorporated utility decrements (derived directly from time trade-off/standard gamble/expert elicitation methods or indirectly using a health-related quality of life questionnaire like the EQ-5D). Specific populations considered included patients receiving DAPT who had previously had a PCI, CABG or ACS patients receiving medication only. Studies assessing antiplatelet monotherapy in these populations were excluded. Studies reporting HRQoL information from which utility decrements could not be derived (e.g., condition-specific, non-preference based HRQoL questionnaires) were excluded.

#### Information sources, search and data collection

Two databases (Ovid MEDLINE and PubMed) were searched from inception to July 23, 2018 (Additional file 1: Appendix A). Search terms were developed for three categories: coronary interventions, DAPT nomenclature and HRQoL terminology. In addition, a hand search of references from included articles was conducted. One author (B.D.) screened the titles and abstracts of all the citations identified from the search strategies, reviewed the full-text articles identified after screening and extracted the data from the included studies.

#### Data items and synthesis of results

The synthesis of the literature search results was stratified by study type (primary research or decision analytic model). Study design, patient population, DAPT regime, categorisation of bleeding, HRQoL instrument and valuation approach used to estimate health state utility values and utility decrements for minor, major and other bleeds reported were extracted. It is quite common for utility decrements to be reported in decision analytic modelling studies with no more than a citation provided and no additional details as to how the decrements were derived. In such cases, the cited references were also reviewed to extract information on the derivation methods. The quality and relevance of the utility decrements identified in each included study was assessed using the checklist outlined by Ara, et al. [20]. Note that as part of the checklist the utility decrements were assess for their adherence to reimbursement agency requirements specifically using the NICE reference case [15].

### Patient elicitation exercise using vignettes and EQ-5D

#### Study design, recruitment and participants

The elicitation exercise was a standalone study conducted alongside a qualitative study involving focus groups that explored the behaviour of patients who had received DAPT after a coronary intervention or an acute coronary event. The focus groups discussed information seeking, medication changes and adherence to medications. The elicitation exercise and qualitative study were run on the same days by different researchers, using the same participant sample. The qualitative study did not include any discussion of the elicitation exercise and therefore its findings are not discussed here.

The participant sample was composed of four separate groups (two groups each with patients exposed to DAPT for either ≤6 or > 6 months), ranging from three to seven participants. The target sample size was ten participants per group (total 40 participants). This was a pragmatic decision aimed to maximise the number of respondents for the elicitation exercise, but given the participant sample was shared the number of participants and required number of groups had to also be manageable for the qualitative study. Potential participants were identified from hospital wards pre-discharge and hospital theatre/catheter laboratory lists. Of the 150 individuals eligible for inclusion and approached by telephone, 68 were invited to participate in the study, of which 37 agreed to participate and received a participant information leaflet, however, 16 did not attend their assigned group session resulting in a sample size of 21 participants (Additional file 1: Appendix B). The 21 participants who attended the group sessions were, however, representative of the eligible pool of patients from which they were drawn (Additional file 1: Appendix C). That is, demographics and treatment characteristics were broadly similar between those who were invited to participate in the study, but did not attend (*n* = 47) and those who did attend a group session (*n* = 21).

#### Data collection

Participants were randomly allocated a colour-coded study booklet (Additional file 1: Appendix D), containing a patient-demographics questionnaire and one of four sequences of six EQ-5D questionnaires and associated vignettes (Table 1). The sequence of the EQ-5D questionnaires and vignettes were varied to avoid ordering effects in participants’ responses. Study booklet allocation used a randomisation scheme with block sizes of two, four and six, stratified by duration of DAPT exposure (≤6 or > 6 months).

**Table 1:** Different sequences of the six EQ-5D questionnaires for the patient elicitation exercise

Sequence Number	Order of the questionnaires					
	1st questionnaire	2nd questionnaire	3rd questionnaire	4th questionnaire	5th questionnaire	6th questionnaire
1	EQ-5D-3 L Baseline	EQ-5D-5 L Baseline	EQ-5D-3 L Vignette A	EQ-5D-3 L Vignette B	EQ-5D-5 L Vignette A	EQ-5D-5 L Vignette B
2	EQ-5D-5 L Baseline	EQ-5D-3 L Baseline	EQ-5D-5 L Vignette A	EQ-5D-5 L Vignette B	EQ-5D-3 L Vignette A	EQ-5D-3 L Vignette B
3	EQ-5D-3 L Baseline	EQ-5D-5 L Baseline	EQ-5D-3 L Vignette B	EQ-5D-3 L Vignette A	EQ-5D-5 L Vignette B	EQ-5D-5 L Vignette A
4	EQ-5D-5 L Baseline	EQ-5D-3 L Baseline	EQ-5D-5 L Vignette B	EQ-5D-5 L Vignette A	EQ-5D-3 L Vignette B	EQ-5D-3 L Vignette A

Participants first completed the demographics and baseline EQ-5D-3 L and EQ-5D-5 L questionnaires as they pertained to their health on that day. As the EQ-5D-3 L is the NICE recommended instrument for assessing the HRQoL impacts of interventions its inclusion allowed our derived decrements to constitute potential evidence for future cost-effectiveness analyses conducted in the UK. Inclusion of the EQ-5D-5 L allowed us to compare the magnitude of utility decrements derived from different EQ-5D questionnaires. Participants then completed EQ-5D-3 L and EQ-5D-5 L modified questionnaires in relation to two vignettes describing minor (Vignette A) and major (Vignette B) bleeds (Additional file 1: Appendix D). Modified versions of the EQ-5D questionnaires were approved by the EuroQoL Research Foundation on June 21, 2017 and used to improve the clarity of the elicitation exercise (e.g., questionnaires completed in relation to vignettes rather than the respondent’s “own” health) and to minimise the burden on participants (e.g., removal of the Visual Analogue Scale). Vignettes were used because there are few opportunities to administer HRQoL questionnaires to patients experiencing bleeds. Patients may not seek medical care for minor bleeds, precluding researchers from interacting with patients at the time of event and major bleeds often represent medical emergencies incapacitating patients.

The vignettes were developed based on the Bleeding Academic Research Consortium (BARC) definitions, [21] which provided standardised nomenclature to differentiate the descriptions of minor (i.e., a bleed that doesn’t result in patients seeking medical care) and major (i.e., a bleed that does result in patients seeking medical care) bleeds. Both vignettes were also reviewed for face validity and updated based on feedback received from two clinicians (a general practitioner and cardiologist). For each vignette, participants completed both the EQ-5D-3 L and EQ-5D-5 L. All participants completed each of the questionnaires individually and did not discuss their answers with other participants. At the bottom of each EQ-5D questionnaire, a supplementary question asked how long participants expected their HRQoL to be affected by the bleed described in the vignette. We expected that this information would be poorly quantified in the literature, yet this information is essential to estimate appropriate utility decrements (i.e., it is required to standardise the loss in HRQoL estimated from the EQ-5D for a specific time period). Therefore, we sought to directly quantify values by asking study participants. It should also be noted that many of the participants (48%; 10/21) reported previously experiencing a minor bleed while on DAPT during the focus group interviews and research has shown that most patients who have or are currently receiving DAPT are cognisant of the range of bleeding risks associated with DAPT [22]. It is therefore likely that all participants would have actively considered the risk of bleeding separately from the elicitation exercise, thus making them suitable surrogates to comment on the impact of bleeding on HRQoL.

#### Missing data and extreme values

As the elicitation exercise was conducted in small groups with oversight from at least one study coordinator, missing data was anticipated to be minimal. Due to the open-ended nature of the supplementary questions there was the potential for participants to report extreme values relative to other participants (the limit for defining an extreme value was a difference of greater than six months or one year from the next closest reported value for minor and major bleeds respectively) or nonsensical values (e.g., HRQoL time impact greater for minor bleed than major bleeds). In such scenarios, we planned to consider reported values as missing and substitute mean values.

#### Data analysis

Responses to the EQ-5D questionnaires were used to estimate mean utility decrements for both minor and major bleeds. Responses were converted to health state utility values using the UK EQ-5D-3 L tariff, [23] UK EQ-5D-5 L tariff, [24] and UK EQ-5D-5 L crosswalk to UK EQ-5D-3 L value set [25]. The latter uses a mapping function to convert EQ-5D-5 L responses to health state utility values from the EQ-5D-3 L tariff. Utility decrements were then derived using linear regression as the primary analysis. EQ-5D-3 L or EQ-5D-5 L utility values associated with either Vignette A or Vignette B were the dependent variable adjusted for baseline EQ-5D utility value, age, sex, coronary intervention received (PCI, CABG or ACS with medical management) and days since commencing DAPT therapy. Control groups were created by duplicating baseline utility values and assuming these values represented hypothetical participants not experiencing a bleed. The regression coefficient for the variable indicating the presence/absence of a bleed represented the mean utility decrement if the effects on HRQoL were to persist for one-year. Using responses from the supplementary questions, the regression coefficients of the bleeding event identifier variables were multiplied by the mean number of days the event was predicted to affect HRQoL and the product divided by 365 days.

An alternative approach to estimating utility decrements was used in a sensitivity analysis to test the robustness of the decrements derived from the primary analysis. By subtracting the utility values for Vignette A or B from a value of one (perfect health), a utility decrement for a bleed if the effects on HRQoL were to persist for one-year for each participant was estimated. Adjustments were made by multiplying these values by the mean number of days the event was predicted to affect HRQoL (derived from the supplementary questions) and dividing the product by 365 days. The mean decrements for the two bleed types were then determined. Note that the calculation approach used in the sensitivity analysis will exaggerate the utility decrement for any patient not otherwise describing their health as perfect and was used to identify maximum plausible values for the minor and major bleeding utility decrements.

Utility decrements from the primary analysis for each EQ-5D questionnaire were compared to each other as well as to decrements from the sensitivity analysis and estimates from our literature review. As it is likely that existing utility decrements identified in our literature review might have been derived for use in cost-effectiveness analyses from the US perspective, responses to the EQ-5D-3 L and EQ-5D-5 L were also converted to health state utility values using the US EQ-5D-3 L tariff [26] and the US EQ-5D-5 L crosswalk to US EQ-5D-3 L value set [25]. The primary and sensitivity analyses were repeated and the results compared to utility decrements identified in our literature review.

## Results

### Literature review

#### Study selection

**Fig. 1:**
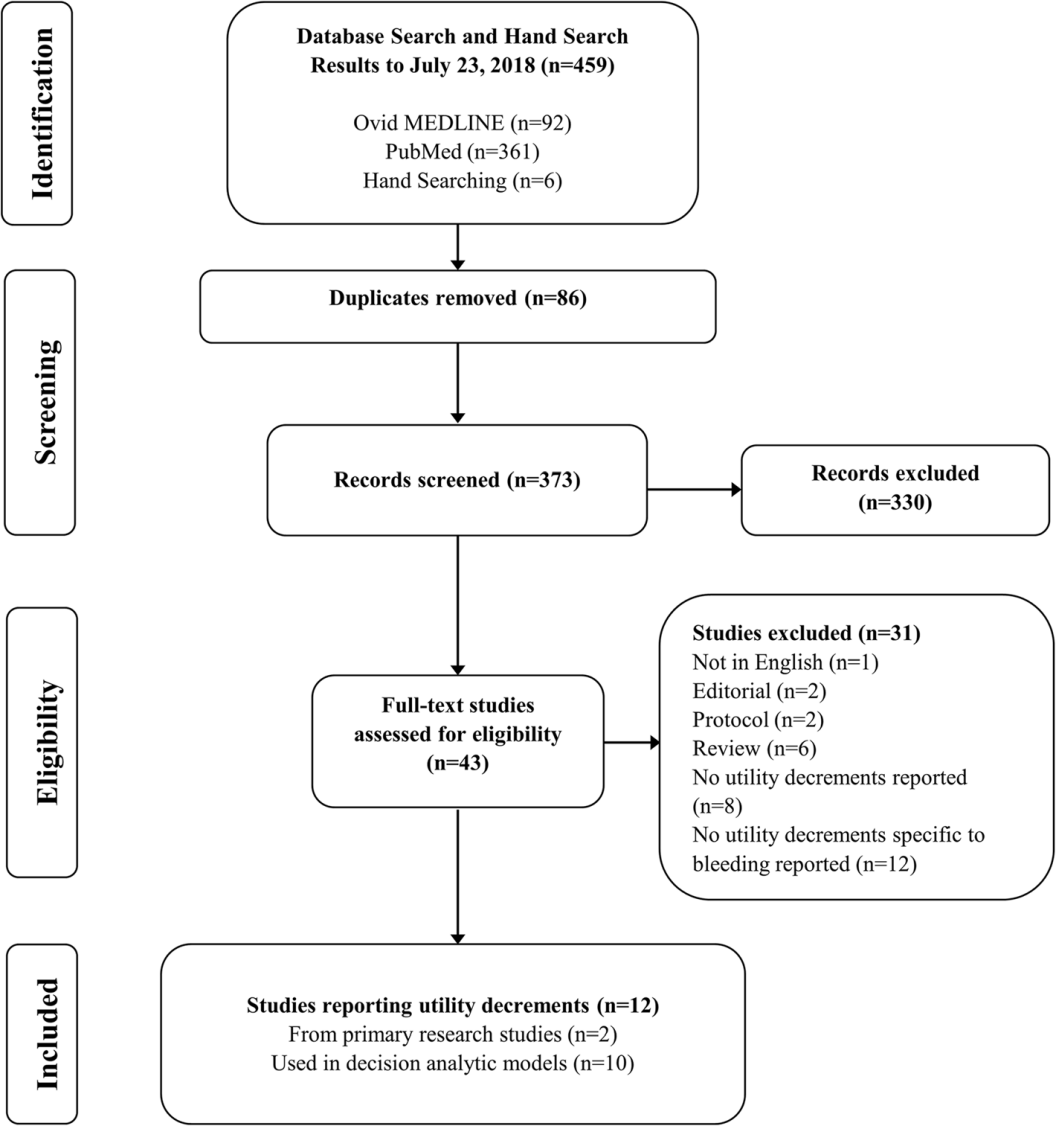
Flow diagram for selection of studies

We identified a total of 459 citations. After removing duplicates (*n* = 86), 373 unique titles and abstracts were screened. Of these, 330 were excluded and 43 were reviewed in full-text. Twelve studies were judged eligible and included in the review (Fig. 1).

#### Existing utility decrements

The 12 eligible studies comprised two primary research studies [10, 11] and ten decision analytic modelling studies [27–36] (Tables 2 and 3 respectively). Utility decrements from the primary research studies, derived using differences in baseline and six-month follow-up responses from the EQ-5D-3 L, ranged from − 0.0257 (95% CI -0.0365 to − 0.0148) for minor bleeds to − 0.0445 (95% CI -0.073 to − 0.016) for major bleeds (Table 2). Utility decrements from decision analytic models ranged from − 0.002 to − 0.02 for minor bleeds and − 0.007 to − 0.05 for major bleeds. Utility decrements were also reported for general bleeding terms such as ‘gastrointestinal bleeds’ ranging from − 0.005 to − 0.016 and decrements of − 0.01, − 0.02, − 0.03, − 0.13 and − 0.25 were reported for ‘CABG-related’, ‘bleeding in general’, ‘extra-cranial’, ‘serious’ and ‘non-fatal bleeds’ respectively (Table 3). A summary of the sources of utility decrements reported in decision analytic models is provided in Additional file 1: Appendix E.

**Table 2:** Utility decrements for bleeding events during dual antiplatelet therapy from primary research studies

Author Country [ref]	Study design	Patient population	Antiplatelet regime	Definition and categorisation of bleeding	Instrument used to measure QoL	Valuation method	Utility decrements for any bleed	Utility decrements for minor bleeds	Utility decrements for major bleeds
Amin US [10]	Prospective, multicentre cohort study (TRIUMP)	3560 AMI patients who had been hospitalised	DAPT post AMI (84.9% and 13% of patients that had a nuisance bleed at any time point received thienopyridine and warfarin respectively at discharge)	Nuisance bleeding (BARC type 1^b^), as the occurrence of any of the four bruising/bleeding events^c^ that did not lead to: hospitalisation, blood transfusion or change of medications by a physician	EQ-5D VAS at baseline, 1, 6 and 12 months	VAS	NR	BARC type 1: − 2.81 (95% CI 1.09 to 5.64) for VAS at 1 month	NR
Amin US [11]	Prospective, observational, longitudinal, multicentre registry (TRANSLATE -ACS)	9,290^a^ AMI patients treated with PCI	DAPT post PCI (clopidogrel in 68%, prasugrel in 29% and ticagrelor in 2%)	Any bleeding or severe bruising event that was patient-reported, associated with an antiplatelet medication change, or independently adjudicated bleeding rehospitalisation based on medical record review; BARC^b^	EQ-5D-3 L questionnaire to calculate index score and VAS at baseline and 6 months	D1 valuation model [26] for index score and direct valuation using VAS	Bleeding associated with a change of − 0.033 (95% CI -0.041 to − 0.026) in index score and − 2.5 (95% CI -3.3 to − 1.8) in VAS	BARC type 1 vs none: − 0.0257 (95% CI -0.0365 to − 0.0148) for index score; − 2.04 (95% CI -3.15 to − 0.093) for VAS	BARC type 2–4 vs none: − 0.0381 (95% CI -0.047 to − 0.0293) for index score; − 2.79 (95% CI -3.70 to − 1.88) for VAS BARC type 3–4 vs none: − 0.0445 (95% CI -0.073 to − 0.016) for index score; − 7.10 (95% CI -10.04 to − 4.16) for VAS

*AMI* acute myocardial infarction, *BARC* Bleeding Academic Research Consortium, *CI* confidence interval, *DAPT* dual antiplatelet therapy, *NR* not reported, *PCI* percutaneous coronary intervention, *QoL* quality of life, *TRANSLATE-ACS* Treatment With Adenosine Diphosphate Receptor Inhibitors: Longitudinal Assessment of Treatment Patterns and Events After Acute Coronary Syndrome, *TRIUMP* Translational Research Investigating Underlying Disparities in Acute Myocardial Infarction Patients’ Health Status, *VAS* visual analog scale

^a^Started with 11,649 patients and excluded those who died in hospital (*n* = 13) or by 6 months (*n* = 106), those with missing baseline (*n* = 76) or 6-month EQ-5D data (*n* = 1928) and those with incomplete medical records or whose hospitalisation events could not be validated (*n* = 236)

^b^See Mehran et al. [21] for the definitions of the nine BARC bleeding types (type 0, type 1, type 2, type 3a, type 3b, type 3c, type 4, type 5a and type 5b)

^c^Nuisance bleeding was assessed vis the following four questions: “Since leaving the hospital after your heart attach, have you had: 1) easy or significant bleeding?; 2) significant bruising?; 3) gum bleeds or nose bleed?; or 4) serious bleeding?”

**Table 3:** Utility decrements for bleeding events during dual antiplatelet therapy from decision analytic models

Author [ref]	Hypothetical patient population modelled	Antiplatelet regime	Definition and categorisation of bleeding	Instrument and population used to measure QoL	Valuation method	Utility decrements for minor bleeds	Utility decrements for major bleeds	Utility decrements for other bleeds
Greenhalgh [27]	Four subgroups: ACS with PCI for STEMI with and without T2DM; and ACS with PCI for UA or NSTEMI with and without T2DM	DAPT – prasugrel plus low-dose aspirin compared to clopidogrel plus low-dose aspirin	MS model definition for bleed does not exclude CABG-related bleeds; non-fatal bleeds not treated as on-going health states within model [such events incur only temporary reduction (14 days) in HRQoL]	MS: EQ-5D-3 L; UK population norms	MS: Time trade-off techniques	NR	MS: 25% decrement to UK population norms (free of disease) for 14 days; equal to a disutility of − 0.007	NR
Garg [28]	ACS with PCI (i.e., DES)	DAPT - clopidogrel plus low-dose aspirin; durations of 12 and 30 months	Major and minor bleeds based on TIMI bleeding risk score [disutility applied during the year in which event occurred]	NR – see Additional file 1: Appendix E for more details	NR – see Additional file 1: Appendix E for more details	−0.002	− 0.025	NR
Kazi [29]	ACS with PCI	Five strategies: 1) generic clopidogrel; 2) prasugrel; 3) ticagrelor; 4) CYP2C19 carriers ticagrelor and noncarriers clopidogrel; 5) CYP2C19 carriers prasugrel and noncarriers clopidogrel	Minor haemorrhage and CABG-related bleeding based on TIMI bleeding risk score and extracranial haemorrhage based on TIMI score	NR – see Additional file 1: Appendix E for more details	NR – see Additional file 1: Appendix E for more details	0.2 for 2 days (− 0.004)	NR	Extra-cranial: 0.2 for 14 days (− 0.0308) CABG-related bleed: 0.5 for 7 days (− 0.01)
Liew [30]	ACS (trial data used included patients scheduled to undergo medical or invasive management (e.g., PCI or CABG)	DAPT – ticagrelor plus aspirin compared to clopidogrel plus aspirin	No clinical definitions reported [disutilities applied during the cycle (1-year cycle length) in which the event occurred]	EQ-5D-3 L	NR	−0.02	− 0.05	NR
Gupta [31]	CAS with PCI receiving either DES or BMS	DAPT - clopidogrel plus aspirin	GI bleeding	Based on the average duration of hospitalisation	NA	NR	NR	GI haemorrhage: toll of 6 days (− 0.016)
Schleinitz [32]	High-risk ACS: unstable angina and electrocardiographic changes or non-Q-wave MI	DAPT – clopidogrel plus aspirin compared to aspirin alone	GI bleeding	Assumption	NA	NR	NR	GI bleeding: − 0.005
Latour-Perez [33]	NSTEMI ACS with hospital admission	DAPT - clopidogrel plus aspirin compared to aspirin alone	GI bleeding [disutility only counted in the cycle (1-month cycle length) in which it occurred]	NR – see Additional file 1: Appendix E for more details	NR – see Additional file 1: Appendix E for more details	NR	NR	Serious haemorrhage disutility − 0.13; GI bleeding referred to in methods section, but no associated disutility value reported
Jiang [34]	ACS with PCI	DAPT – Three strategies: 1) clopidogrel plus aspirin; 2) prasugrel or ticagrelor plus aspirin; and 3) CYP2C19 LOF/GOF allele prasugrel or ticagrelor plus aspirin and wild type clopidogrel plus aspirin	Nonfatal bleeding	NR – see Additional file 1: Appendix E for more details	NR – see Additional file 1: Appendix E for more details	NR	NR	Nonfatal bleeding: −0.250
Wang [35]	60-year old Chinese (North Asian) ACS patients who underwent PCI	DAPT – Three strategies: 1) clopidogrel plus aspirin; 2) ticagrelor plus aspirin; and 3) CYP2C19*2 allele carriers receive ticagrelor plus aspirin and wild type clopidogrel plus aspirin	Bleeding	NR – see Additional file 1: Appendix E for more details	NR – see Additional file 1: Appendix E for more details	NR	NR	Bleeding: − 0.02
Jiang [36]	60-year old ACS patients undergoing PCI	DAPT – Four strategies: 1) clopidogrel plus aspirin; 2) prasugrel or ticagrelor plus aspirin; 3) CYP2C19 LOF/GOF allele prasugrel or ticagrelor plus aspirin and wild type clopidogrel plus aspirin; and 4) low responders (PRU > 208) clopidogrel loading dose followed by prasugrel or ticagrelor plus aspirin and normal responders (PRU ≤ 208) clopidogrel plus aspirin.	Nonfatal bleeding	NR – see Additional file 1: Appendix E for more details	NR – see Additional file 1: Appendix E for more details	NR	NR	Nonfatal bleeding: −0.250

*ACS* acute coronary syndrome, *AF* atrial fibrillation, *AG* assessment group, *BMS* bare metal stent, *CABG* coronary artery by-pass grafting, *CAS* coronary artery stenosis, *DAPT* dual antiplatelet therapy, *DES* drug-eluting stent, *GI* gastrointestinal, *GOF* gain-of-function, *HRQoL* health-related quality of life, *ICD-9* International Statistical Classification of Diseases and Related Health Problems Version 9, *LOS* loss-of-function, *MI* myocardial infarction, *MS* manufacturer’s submission, *NA* not applicable, *NR* not reported, *NSTEMI* non-ST segment elevation myocardial infarction, *PAD* peripheral arterial disease, *PCI* percutaneous coronary intervention, *PRU* P2Y_12_ reaction units, *QoL* quality of life, *SD* standard deviation, *SG* standard gamble, *STEMI* ST segment elevation myocardial infarction, *T2DM* type 2 diabetes mellitus, *TIMI* [28], *UA* unstable angina

#### Quality and relevance assessment

Based on the information provided in the text of the included studies as well as in associated references, the results of our quality and relevance assessment is provided in Additional file 1: Appendix F. Overall, the utility decrements for bleeding events from the included studies were derived mainly from studies with limited relevance to the population of interest and lacked comprehensive reporting to accurately assess their risk of bias. Only half the studies provided adequate details concerning the measurement and valuation of the reported utility decrements and all the included studies were not completely aligned with reimbursement agency requirements in the UK.

### Patient elicitation exercise using vignettes and EQ-5D

#### Baseline patient characteristics

Participants were elderly (mean age 66), most were male (20/21) and all participants reported their ethnicity as White British (21/21) (Table 4). The majority had received PCI (14/21) with others receiving CABG (6/21) or medical management due to ACS (1/21). DAPT exposure time was ≤6 and > 6 months for nine and 12 of the participants respectively. Baseline EQ-5D health state utility values ranged from 0.760 on the EQ-5D-3 L UK tariff and EQ-5D-5 L to EQ-5D-3 L crosswalk value set to 0.833 on the EQ-5D-5 L UK tariff.

**Table 4:** Baseline participant characteristics from the elicitation exercise

Characteristic	Label	Mean (range or %)
Age (*n* = 21)		66.3 (48 to 88)
Sex (*n* = 21)	Female Male	1 (5%) 20 (95%)
Ethnicity (*n* = 21)	White British	21 (100%)
Coronary intervention received (*n* = 21)	PCI CABG Medical Management	14 (67%) 6 (29%) 1 (5%)
DAPT exposure time (*n* = 21)	≤6 months	9 (43%)
	> 6 months	12 (57%)
Months received DAPT (*n* = 20)^a^		7.5 (0 to 15)
Days since starting DAPT (*n* = 21)^b^		235 (18 to 561)
Reported previously experiencing a minor bleed while on DAPT^c^		10 (48%)
EQ-5D-3 L UK tariff (*n* = 21)		0.760 (0.159 to 1)
EQ-5D-3 L US tariff (*n* = 21)		0.816 (0.446 to 1)
EQ-5D-5 L UK tariff (*n* = 21)		0.824 (0.197 to 1)
EQ-5D-5 L UK crosswalk (*n* = 21)		0.760 (0.221 to 1)
EQ-5D-5 L US crosswalk (*n* = 21)		0.817 (0.440 to 1)

*CABG* coronary artery bypass grafting, *DAPT* dual antiplatelet therapy, *PCI* percutaneous coronary intervention

^a^This is self-reported from the patient-demographics questionnaire; a value of zero for this variable indicates that the participant had received DAPT for less than a month

^b^Days between the date of the focus group and the date the participant commenced DAPT therapy. The date the participant commenced DAPT therapy was derived from the screening questionnaire used during recruitment

^c^This information was not collected in the patient-demographics questionnaire, but rather ascertained in the discussions that occurred during the qualitative interviews that were conducted as part of a separate study using the same participant sample that completed the elicitation exercise

#### Missing data and extreme values

All but one participant (20/21) completed the demographics questionnaire with no missing data; the remaining participant did not report the number of months over which they had taken DAPT. The two baseline EQ-5D questionnaires were completed with no missing data. Complete data were obtained for the EQ-5D-3 L for both Vignettes A and B; one participant did not complete the EQ-5D-5 L for either Vignette A or B; and one participant only responded to the pain and anxiety/depression domains for the EQ-5D-5 L for Vignette A. In addition, five participants did not respond to the supplementary question (i.e., duration of decrement in HRQoL) for both Vignette A and B with the EQ-5D-3 L; missing values were imputed with mean values of 7.60 and 45.38 days respectively. Five and four participants did not respond to this question for Vignette A and B respectively with the EQ-5D-5 L; missing values were imputed with mean values of 10.93 and 48.75 days respectively.

One participant reported extreme values of ten years for Vignette A and four years for Vignette B for the EQ-5D-5 L (next closest values were three and ten months respectively), which is perhaps counterintuitive given Vignette A represents a less severe health state (minor bleed) compared to Vignette B (major bleed). The same participant also reported an extreme value of one year for Vignette A (next closest value was three months) and no response for Vignette B for the EQ-5D-3 L. These three extreme values were set to missing and imputed with the respective mean value.

#### Utility decrements for minor and major bleeding events

Utility decrements for both minor and major bleeding events derived using linear regression (primary analysis) and the alternative approach (sensitivity analysis) are presented in Table 5. For the primary analysis, the utility decrements estimated using the two EQ-5D questionnaires and different valuation methods are relatively similar (range − 0.000848 to − 0.00250 for minor bleeds and − 0.0187 to − 0.0297 for major bleeds). The EQ-5D-3 L UK tariff resulted in the largest utility decrement for both minor and major bleeds (− 0.00250 and − 0.0297 respectively). Applying the US tariff to the EQ-5D-3 L resulted in slightly smaller decrements (− 0.00180 and − 0.0203). The EQ-5D-5 L UK tariff resulted in the smallest utility decrement for minor bleeds (− 0.000848) and a smaller utility decrement for major bleeds when compared to the respective value for the EQ-5D-3 L UK tariff (0.0222 versus 0.0297). Utility decrements derived from crosswalk values were smaller than the values estimated from the EQ-5D-3 L using both the UK and US tariffs for both major and minor bleeds (Table 5). Complete regression results are provided in Additional file 1: Appendix G.

**Table 5:** Utility decrements for minor and major bleeding events using regression-based approach (primary analysis) and alternative approach (sensitivity analysis); mean (standard deviation)

Instrument	Primary Analysis		Sensitivity Analysis	
	Minor Bleed^a^	Major Bleed^b^	Minor Bleed^c^	Major Bleed^d^
EQ-5D-3 L UK tariff (*n* = 21)	− 0.00250 (0.00265)	− 0.0297 (0.0478)	− 0.00828 (0.0155)	− 0.0621 (0.103)
EQ-5D-3 L US tariff (*n* = 21)	− 0.00180 (0.00190)	− 0.0203 (0.0328)	− 0.00584 (0.0102)	− 0.0441 (0.0705)
EQ-5D-5 L to EQ-5D-3 L UK value set (*n* = 19; *n* = 20)^e^	− 0.00140 (0.00280)	−0.0258 (0.0421)	− 0.00661 (0.00911)	−0.0552 (0.0830)
EQ-5D-5 L to EQ-5D-3 L US value set (*n* = 19; *n* = 20)^e^	− 0.00137 (0.00275)	−0.0187 (0.0305)	− 0.00566 (0.00880)	− 0.0405 (0.0597)
EQ-5D-5 L UK tariff (*n* = 19; *n* = 20)^e^	− 0.000848 (0.00170)	− 0.0222 (0.0362)	− 0.00453 (0.00614)	−0.0465 (0.0700)

^a^Utility decrements obtained by multiplying the regression coefficient for the bleeding event identifier variable by the mean number of days (7.60 days for EQ-5D-3 l and 10.93 for EQ-5D-5 L) that a minor bleed is expected to affect health-related quality-of-life and dividing the product by 365 days

^b^Utility decrements obtained by multiplying the regression coefficient for the bleeding event identifier variable by the mean number of days (45.38 days for EQ-5D-3 L and 48.75 for EQ-5D-5 L) that a major bleed is expected to affect health-related quality-of-life and dividing the product by 365 days

^c^Utility decrements obtained by subtracting the health state utility value associated with Vignette A (minor bleed) from one (perfect health) and multiplying by the mean number of days (7.60 days for EQ-5D-3 l and 10.93 for EQ-5D-5 L) that a minor bleed is expected to affect health-related quality-of-life and dividing the product by 365 days

^d^Utility decrements obtained by subtracting the health state utility value associated with Vignette B (major bleed) from one (perfect health) and multiplying by the mean number of days (45.38 days for EQ-5D-3 L and 48.75 for EQ-5D-5 L) that a major bleed is expected to affect health-related quality-of-life and dividing the product by 365 days

^e^One participant did not complete the EQ-5D-5 L for either Vignette A or B and one participant only responded to the pain and anxiety domains for the EQ-5D-5 L for Vignette A resulting in two missing values for minor bleeds and one missing value of major bleeds

#### Sensitivity analysis

Using the alternative estimation approach resulted in utility decrements that were larger compared to the values estimated in the primary analysis (range 0.00453 to 0.00828 for minor bleeds and 0.0405 to 0.0621) (Table 5). The relative magnitude of the utility decrements followed the same pattern as observed in the primary analysis. For both minor and major bleeds, the largest difference between the utility decrements estimated in the primary and sensitivity analyses was for the EQ-5D-3 L UK tariff (difference of 0.00578 and 0.0324 respectively).

#### Comparing utility decrements from all sources

An ordering by magnitude of the derived and existing utility decrements for minor and major bleeds is presented in Table 6. For minor bleeds the utility decrements range from − 0.000848 to − 0.0257. Whereas, for major bleeds the utility decrements ranged from − 0.005 to − 0.250.

**Table 6:** Derived and existing utility decrements for minor and major bleeds ordered by magnitude

Source	Utility decrement for minor bleeds
EQ-5D-5 L UK tariff - PA	−0.000848
EQ-5D-5 L to EQ-5D-3 L US value set - PA	−0.00137
EQ-5D-5 L to EQ-5D-3 L UK value set - PA	−0.00140
EQ-5D-3 L US tariff - PA	−0.00180
Garg [28]	− 0.002
EQ-5D-3 L UK tariff - PA	−0.00250
Kazi [29]	−0.004
EQ-5D-5 L UK tariff - SA	−0.00453
EQ-5D-5 L to EQ-5D-3 L US value set - SA	−0.00566
EQ-5D-3 L US tariff - SA	−0.00584
EQ-5D-5 L to EQ-5D-3 L UK value set - SA	−0.00661
EQ-5D-3 L UK tariff - SA	−0.00828
Liew [30]	− 0.02
Amin [11]	−0.0257 (BARC type 1)
Source	Utility decrement for major bleeds
Schleinitz [32]	−0.005 (GI bleeding)
Greenhalgh [27]	− 0.007
Kazi [29]	−0.01 (CABG-related)
Gupta [31]	−0.016 (GI haemorrhage)
EQ-5D-5 L to EQ-5D-3 L US value set - PA	−0.0187
Wang [35]	− 0.02 (bleeding in general)
EQ-5D-3 L US tariff - PA	−0.0203
EQ-5D-5 L UK tariff - PA	−0.0222
Garg [28]	− 0.025
EQ-5D-5 L to EQ-5D-3 L UK value set - PA	−0.0258
EQ-5D-3 L UK tariff - PA	−0.0297
Kazi [29]	− 0.0308 (extra-cranial)
Amin [11]	−0.0381 (BARC type 2–4)
EQ-5D-5 L to EQ-5D-3 L US value set - SA	−0.0405
EQ-5D-3 L US tariff - SA	−0.0441
Amin [11]	− 0.0445 (BARC type 3–4)
EQ-5D-5 L UK tariff - SA	− 0.0465
Liew [30]	−0.05
EQ-5D-5 L to EQ-5D-3 L UK value set - SA	−0.0552
EQ-5D-3 L UK tariff - SA	−0.0621
Latour-Perez [33]	− 0.13 (serious haemorrhage)
Jiang [34]	−0.250 (non-fatal bleeding)
Jiang [36]	−0.250 (non-fatal bleeding)

*BARC* Bleeding Academic Research Consortium, *CABG* coronary artery bypass grafting, *GI* gastrointestinal, *PA* primary analysis, *SA* sensitivity analysis

## Discussion

The evidence of utility decrements for bleeds in patients receiving DAPT after coronary interventions is limited. Data sources used to estimate utility decrements lack relevance to the population of interest and have been inadequately reported, precluding an accurate assessment of their susceptibility to bias. Adequate details of measurement and valuation are only provided for half the studies and no study completely aligned with reimbursement agency requirements in the UK according to the NICE reference case. The highest quality evidence was reported by Amin et al., [11] but this study used a US population, applying the EQ-5D-3 L US tariff (limits generalisability to other jurisdictions). The decrements were also based on differences in HRQoL estimated over six-months, which is an overestimation of the length of time a bleed would affect HRQoL compared to responses from the supplementary questions in our study (8–11 days and 45–49 days for minor and major bleeds respectively). Our primary research study attempted to elicit the length of time a bleed would affect HRQoL from patients who either had experienced a minor bleed or were highly likely to have actively considered the risk of bleeding outside the elicitation exercise, whereas existing studies have based this length of time on clinical assumptions or used the time difference between study follow-up points.

Utility decrements derived from the patient elicitation exercise were consistent with some of the existing estimates (Table 6). The utility decrement for minor bleeds estimated from the EQ-5D-3 L UK tariff in the primary analysis of our study (− 0.00250) is similar to decrements reported by Garg et al. [28] and Kazi et al. [29] (− 0.002 and − 0.004 respectively) that were both based on an unclear synthesis of values reported from the consensus of three internists [37] and a direct elicitation using standard gamble methods [38]. In contrast, there is a large difference in the decrements estimated from the EQ-5D-3 L US tariff in the primary and sensitivity analysis for our study (− 0.00180 and − 0.00584 respectively) and the decrement reported by Amin et al. [11] who also used the EQ-5D-3 L US tariff (− 0.0257). In comparison to EQ-5D-3 L US tariff utility decrements for other conditions [39] the utility decrement for minor bleeding reported by Amin et al. seems large. Similar decrements are reported for mononeuritis of the upper limb (− 0.0244), chronic ulcer of the skin (− 0.0272) and migraine (− 0.0297). These conditions would seem to be associated with greater HRQoL affects compared to minor bleeds, that by the BRAC definition do not cause patients to seek treatment. In contrast, the utility decrements for minor bleeds derived in our study are comparable to decrements reported for chronic sinusitis (− 0.0022) and other dental disorders (− 0.003), which would likely have a similar effect on HRQoL as minor bleeds.

The utility decrements for major bleeds estimated from the EQ-5D-3 L and EQ-5D-5 L using the UK tariffs in the primary analysis of our study (− 0.0297 and − 0.0222 respectively) are similar to decrements reported by Garg et al. [28] and Kazi et al. [29] (− 0.025 and − 0.0381 respectively). Decrements estimated from the EQ-5D-3 L US tariff in the primary and sensitivity analysis for our study (− 0.0203 and − 0.0441 respectively) are similar to the decrements reported by Amin et al. [11] for BARC type 2–4 and type 3–4 bleeds (− 0.0381 and − 0.0445 respectively).

From our elicitation exercise it is apparent that utility decrements estimated from the EQ-5D-3 L are consistently larger than decrements estimated from the EQ-5D-5 L. The differences in decrements were larger when EQ-5D-3 L values were compared to EQ-5D-5 L values directly (differences of 0.00165 and 0.0075 for minor and major bleeds respectively), with small differences observed when EQ-5D-3 L values were compared to values obtained using the EQ-5D-5 L to EQ-5D-3 L crosswalk value set (differences of 0.0011 and 0.0039 respectively). This is not surprising, as the EQ-5D-5 L has been shown to shift mean utility values closer to 1 (full health), compressing them into a smaller range compared to the EQ-5D-3 L [40]. This difference can potentially cause improvements in HRQoL to be valued less when using the EQ-5D-5 L compared to the EQ-5D-3 L, however, the impact of using utility decrements derived from the different versions of the EQ-5D on the cost-effectiveness of DAPT has yet to be elucidated and will be a valuable line of future research.

Our study has several limitations. Firstly, our derived utility decrements are based on responses to the EQ-5D associated with vignettes describing minor and major bleeds and responses from participants estimating the length of time a bleed would impact their HRQoL. Participants completing the elicitation exercise may not have directly experienced a major bleed, but most had previously experienced a minor bleed while on DAPT. All participants were, however, recruited for the study due to their current or past experience taking DAPT and research has shown that most patients on DAPT are aware of the range of bleeding risks associated with DAPT [22]. As such, it is likely that all participants would have been informed of the risk of bleeds while on DAPT by their treating physician, thus, making them suitable surrogates. Furthermore, the are a number existing studies that have successfully employed the vignette approach to eliciting utility values/decrements using participant samples with no first-hand experience or knowledge of the health states they were being asked to value [41–43]. These existing studies have justified the vignette approach as existing evidence was of poor quality and relevance (which we also showed in our review) and that direct measurement in affected patients would be difficult (which is also the case for major and minor bleeds as patients are incapacitated and don’t interact with the healthcare system at the time of the event respectively).

Secondly, our study population was small (*n* = 21) and homogeneous, potentially limiting generalisability. Furthermore, 16 of the 37 participants who agreed to participate in the study did not attend their assigned group session. The reasons for non-attendance are not clear, but could be due to reduced HRQoL, employment status or a greater travel distance to the study location. These potential differences may bias our results, but the direction of such bias is unclear. That being said, our sample is broadly comparable in demographic and treatment characteristics to those individuals who were invited to participant, but did not attend (Additional file 1: Appendix C) as well as to a whole of England PCI registry that reports demographics of 74% male and 90% Caucasian [44]. In addition, given the questionable quality and relevance to the UK context of the existing evidence identified in our review (some decrements derived from expert elicitation of only three medical internists or a single clinician), [28, 29, 33, 35] we believe that our larger sample and applied methods represent an improvement compared to approaches used previously.

Thirdly, the elicitation exercise required cognitive processing that may have been difficult for some participants due to advanced age (some participants were > 80 years old and noticeably fatigued/lost concentration during the 20 min exercise; this was in addition to an hour long group discussion). A few participants commented that it was difficult to imagine that they were the individual described in the vignettes. However, as the groups were small the study coordinators ensured that all of the participants understood the exercise and completed all questionnaires to the best of their ability.

Fourthly, some of the participants reported difficulty in assessing the impact of a major bleed (i.e., a bleed that results in patients seeking medical care) on HRQoL given the range of different examples presented in the vignette (e.g., persistent nose bleed, blood in your bowel movement, vomiting blood or bleeding in your eye). As we were interested in estimating an average utility decrement for a major bleeding event, in general, it was not possible to limit the vignette description to a specific type of bleed. Furthermore, the vignette for major bleeds was developed using the BARC definitions, which encompass several concepts of seriousness when classifying bleeds considered ‘major’ [21]. For the few participants expressing difficulty, guidance from the supervising researcher was provided, indicating that the participant should try to account for all potential impacts of the bleeds described in the vignette in their responses. It is, however, possible that participants limited their responses to the impact of only one of the example bleeds described, but it is not clear if participants would have selected the “less” or more “severe” example bleed in their responses.

Despite the limitations, the patient elicitation exercise provides a clear approach to estimating utility decrements for adverse events that may otherwise be difficult to obtain. For minor bleeds, alternative approaches such as expert elicitation might be less reliable, since clinicians have limited ability to observe the HRQoL impacts of such events as by definition minor bleeds do not cause patients to seek medical care [21]. The elicitation exercise also has the added advantage over direct elicitation approaches like time trade-off [45] or standard gamble, [46] in that it captures both the patients’ understandings of the HRQoL impacts and allows for the use of general population preferences in estimating utility values as recommended by many reimbursement agencies like NICE [15].

Our study has also raised the question of whether the EQ-5D is a suitable instrument to capture HRQoL impacts of adverse events. This was reflected in our study by the confusion experienced by many participants when trying to understand why certain questions of the EQ-5D were relevant to the health state described in the vignettes. For example, one participant stated: “*Why would my ability to walk be affected by a nose bleed?*”. It seemed participants were expecting questions to be directly related to the event described in the vignettes, such as those likely to be included in a preference-based condition-specific measure of HRQoL. It may, therefore, be of interest to explore such HRQoL questionnaires when using the patient elicitation vignette approach.

## Conclusion

Overall, the choice of utility decrement to use in any future cost-effectiveness analysis of DAPT will of course be dependent on the country for which the analysis is being conducted. The utility decrements estimated in our elicitation exercise have been derived from a relevant patient population, based on standardised definitions of minor and major bleeding events, using a validated HRQoL instrument for the patient population of interest and have been valued using general population tariffs. We therefore recommend that our utility decrements (choosing the ones most appropriate for the country to which the analysis will be applied) be used in future cost-effectiveness analyses of DAPT, particularly for minor bleeding events where existing evidence is rather limited. In addition, rather than using a range of alternative sources in cost-effectiveness models, some which may be unreliable, we recommend that future research focuses on quantifying the value of reducing decision uncertainty for our estimated utility decrements. This research would demonstrate whether conducting a larger, more robust study to collect additional information concerning the HRQoL impact of minor and major bleeds for patients taking DAPT post-coronary intervention would be an efficient use of resources.

## Additional file

Additional file 1: Appendicies A to G. (DOCX 266 kb)
