# Supplementary material for: Health-related quality of life impact of minor and major bleeding events during dual antiplatelet therapy: a systematic literature review and patient preference elicitation study
Source: Health Qual Life Outcomes. 2018 Sep 20;16:191. doi: 10.1186/s12955-018-1019-3 (PMC6149200; doi:10.1186/s12955-018-1019-3)
Supplement: Supplementary file 1 — Appendicies A to G. (DOCX 266 kb) [file 12955_2018_1019_MOESM1_ESM.docx]

**appendix a – search strategy**

**Database:** Epub Ahead of Print, In-Process & Other Non-Indexed Citations, Ovid MEDLINER(R) 1946 to Present

**Date of search:** 18/11/2016

1. atrial fibrillation/ or heart arrest/ or myocardial ischemia/ or *acute coronary syndrome/ or coronary disease/ or coronary artery disease/ or *coronary thrombosis/ or *myocardial infarction/ or *thromboembolism/ or *thrombosis/ or "*coronary artery disease"/
2. acute coronary syndrome.ab,hw,kf,kw,ot,sh,ti,tw.
3. myocardial infarction.ab,hw,kf,kw,ot,sh,ti,tw.
4. coronary artery disease.ab,hw,kf,kw,ot,sh,ti,tw.
5. coronary thrombosis.ab,hw,kf,kw,ot,sh,ti,tw.
6. 1 or 2 or 3 or 4 or 5
7. heart bypass, right/ or *angioplasty, balloon, coronary/ or *atherectomy, coronary/ or *coronary artery bypass/ or *angioplasty/ or *angioplasty, balloon/ or *percutaneous coronary intervention/
8. coronary artery bypass grafting.ab,hw,kf,kw,ot,sh,ti,tw.
9. coronary stent.ab,hw,kf,kw,ot,sh,ti,tw.
10. percutaneous coronary intervention.ab,hw,kf,kw,ot,sh,ti,tw.
11. coronary interventions.ab,hw,kf,kw,ot,sh,ti,tw.
12. heart bypass surgery.ab,hw,kf,kw,ot,sh,ti,tw.
13. 7 or 8 or 9 or 10 or 11 or 12
14. platelet aggregation inhibitors/ or aspirin/ or aspirin, dipyridamole drug combination/ or dipyridamole/ or prasugrel hydrochloride/ or exp ticlopidine/
15. antiplatelet therapy.ab,hw,kf,kw,ot,sh,ti,tw.
16. dual antiplatelet therapy.ab,hw,kf,kw,ot,sh,ti,tw.
17. aspirin.ab,hw,kf,kw,ot,sh,ti,nm,tw.
18. clopidogrel.ab,hw,kf,kw,ot,sh,ti,nm,tw.
19. prasugrel.ab,hw,kf,kw,ot,sh,ti,nm,tw.
20. ticagrelor.ab,hw,kf,kw,ot,sh,ti,nm,tw.
21. 14 or 15 or 16 or 17 or 18 or 19 or 20
22. anticoagulants/ or *warfarin/ or *dabigatran/ or *factor xa inhibitors/ or *rivaroxaban/
23. anticoagulant therapy.ab,hw,kf,kw,ot,sh,ti,tw.
24. vitamin k antagonists.ab,hw,kf,kw,ot,sh,ti,tw.
25. triple therapy.ab,hw,kf,kw,ot,sh,ti,tw.
26. warfarin.ab,hw,kf,kw,ot,sh,ti,nm,tw.
27. dabigatran.ab,hw,kf,kw,ot,sh,ti,nm,tw.
28. rivaroxaban.ab,hw,kf,kw,ot,sh,ti,nm,tw.
29. apixaban.ab,hw,kf,kw,ot,sh,ti,nm,tw.
30. 22 or 23 or 24 or 25 or 26 or 27 or 28 or 29
31. 21 and 30
32. exp "quality of life"/ or *comparative effectiveness research/ or *health status indicators/ or *self report/ or exp patient outcome assessment/
33. quality of life.ab,hw,kf,kw,ot,sh,ti,tw.
34. health-related quality of life.ab,hw,kf,kw,ot,sh,ti,tw.
35. health state utility$.ab,hw,kf,kw,ot,sh,ti,tw.
36. multi-attribute utilit$.ab,hw,kf,kw,ot,sh,ti,tw.
37. preference-based measure.ab,hw,kf,kw,ot,sh,ti,tw.
38. quality-adjusted life-years.ab,hw,kf,kw,ot,sh,ti,tw.
39. EQ-5D.ab,hw,kf,kw,ot,sh,ti,tw.
40. SF-6D.ab,hw,kf,kw,ot,sh,ti,tw.
41. HUI-III.ab,hw,kf,kw,ot,sh,ti,tw.
42. AQoL.ab,hw,kf,kw,ot,sh,ti,tw.
43. 32 or 33 or 34 or 35 or 36 or 37 or 38 or 39 or 40 or 41 or 42
44. hemorrhage/ or *ecchymosis/ or *epistaxis/ or *exsanguination/ or *gastrointestinal hemorrhage/ or *gingival hemorrhage/ or *uterine hemorrhage/
45. $bleeding$.ab,hw,kf,kw,ot,sh,ti,tw.
46. 44 or 45
47. 6 and 13 and 21 and 43 – **Total hits: 89**
48. Update 21/11/2016 to 14/08/2017 – **Total hits: 3**
49. Update 21/08/2017 to 23/07/2018 – **Total hits: 0**
50. **Total hits: 92**

**Database:** PubMed

**Date of search:** 28/11/2016

1. (((((((((heart arrest[MeSH Terms]) OR myocardial ischemia[MeSH Terms]) OR acute coronary syndrome[MeSH Terms]) OR coronary artery disease[MeSH Terms]) OR coronary thrombosis[MeSH Terms]) OR myocardial infarction[MeSH Terms]) OR thromboembolism[MeSH Terms]) OR coronary artery disease[MeSH Terms]) OR atrial fibrillation[MeSH Terms]) OR coronary disease[MeSH Terms]
2. acute coronary syndrome[Title/Abstract]
3. myocardial infarction[Title/Abstract]
4. coronary artery disease[Title/Abstract]
5. coronary thrombosis[Title/Abstract]
6. 1 or 2 or 3 or 4 or 5
7. (((((((heart bypass, right[MeSH Terms]) OR heart bypass, left[MeSH Terms]) OR angioplasty, balloon, coronary[MeSH Terms]) OR atherectomy, coronary[MeSH Terms]) OR coronary artery bypass[MeSH Terms]) OR angioplasty[MeSH Terms]) OR angioplasty, balloon[MeSH Terms]) OR angioplasty, transluminal, percutaneous coronary[MeSH Terms]
8. coronary artery bypass grafting[Title/Abstract]
9. coronary stent[Title/Abstract]
10. percutaneous coronary intervention[Title/Abstract]
11. coronary intervention[Title/Abstract]
12. heart bypass surgery[Title/Abstract]
13. 7 or 8 or 9 or 10 or 11 or 12
14. ((((((blood platelet aggregation inhibitors[MeSH Terms]) OR platelet aggregation inhibitors[MeSH Terms]) OR aspirin[MeSH Terms]) OR dipyridamole[MeSH Terms]) OR ticlopidine[MeSH Terms]) OR antiplatelet agents[MeSH Terms]) OR antiplatelet drugs[MeSH Terms]
15. antiplatelet[Title/Abstract]
16. dual antiplatelet therapy[Title/Abstract]
17. aspirin[Title/Abstract]
18. clopidogrel[Title/Abstract]
19. prasugrel[Title/Abstract]
20. ticagrelor[Title/Abstract]
21. 14 or 15 or 16 or 17 or 18 or 19 or 20
22. ((anticoagulant agents[MeSH Terms]) OR anticoagulant drugs[MeSH Terms]) OR warfarin[MeSH Terms]
23. anticoagulant therapy[Title/Abstract]
24. vitamin k antagonists[Title/Abstract]
25. triple therapy[Title/Abstract]
26. warfarin[Title/Abstract]
27. dabigatran[Title/Abstract]
28. rivaroxaban[Title/Abstract]
29. apixaban[Title/Abstract]
30. 22 or 23 or 24 or 25 or 26 or 27 or 28 or 29
31. 21 and 30
32. (((((quality of life[MeSH Terms]) OR comparative effectiveness research[MeSH Terms]) OR index, health status[MeSH Terms]) OR health status indicator[MeSH Terms]) OR assessment, patient outcome[MeSH Terms]) OR life year, quality adjusted[MeSH Terms]
33. quality of life[Title/Abstract]
34. health-related quality of life[Title/Abstract]
35. health state utilit*[Title/Abstract]
36. multi-attribute utilit*[Title/Abstract]
37. preference-based measure[Title/Abstract]
38. quality-adjusted life-year*[Title/Abstract]
39. EQ-5D*[Title/Abstract]
40. SF-6D[Title/Abstract]
41. HUI-III[Title/Abstract]
42. AQoL[Title/Abstract]
43. 32 or 33 or 34 or 35 or 36 or 37 or 38 or 39 or 40 or 41 or 42
44. (((((ecchymosis[MeSH Terms]) OR epistaxis[MeSH Terms]) OR exsanguination[MeSH Terms]) OR gastrointestinal hemorrhage[MeSH Terms]) OR gingival hemorrhage[MeSH Terms]) OR uterine hemorrhage[MeSH Terms]
45. *bleeding*[Title/Abstract]
46. 44 or 45
47. 6 and 13 and 21 and 43 – **Total hits: 321**
48. Update 05/12/2016 to 14/08/2017 – **Total hits: 23**
49. Update 21/08/2017 to 23/07/2018 – **Total hits: 17**
50. **Total hits: 361**

**appendix B – CONSORT Flow Diagram**

**Eligible for inclusion and approached by telephone** (n=150)

**Call answered by patient or family member** (n=82)

**No reply on telephone** (n=68)

**Study explained and patient invited to participate in focus group meetings** (n=68)

**Exclusions** (n=14):

*Patient deceased (n=7); current inpatient (n=4); issues with comprehension (n=1); Alzheimer’s (n=1); not on DAPT (1)*

**Participant Information Leaflet not sent** (n=31):

*Not interested (n=11); no reason (n=9); other commitments (n=5); not happy to be contacted (n=2); lives too far away (n=2); too ill (n=2)*

**Participant Information Leaflet sent** (n=37)

**Exclusions** (n=16):

*Unable to attend meeting (n=12); working (n=1); unwell (n=1); lives too far away (n=1); no reason (n=1)*

**Attended focus group meeting and provided written consent** (n=21)

**appendix C – Comparison of Demographics and Treatment Characteristics of individuals who were invited to participate in the study and either attended or did not attend a focus group Meeting**

|  |  | **Invited to participate, but did not attend (n=47)** | | **Invited to participate and attended (n=21)** | | **Total (n=68)** | |
| --- | --- | --- | --- | --- | --- | --- | --- |
| **Sex; n (%)** | Female | 8/47 | (17%) | 1/21 | (5%) | 9/68 | (13%) |
|  | Male | 39/47 | (83%) | 20/21 | (95%) | 59/68 | (87%) |
| **Age (mean; SD)** |  | 64.5 | (9.4) | 66.3 | (11.3) | 65.0 | (10.0) |
| **Ethnic group** | White | 37/47 | (79%) | 21/21 | (100%) | 58/68 | (85%) |
|  | Asian | 1/47 | (2%) | 0/21 | (0%) | 1/68 | (1%) |
|  | Not recorded | 9/47 | (19%) | 0/21 | (0%) | 9/68 | (13%) |
| **Procedure** | PCI | 27/46 | (59%) | 14/21 | (67%) | 41/67 | (61%) |
|  | CABG | 18/46 | (39%) | 6/21 | (29%) | 24/67 | (36%) |
|  | PCI and CABG | 1/46 | (2%) | 0/21 | (0%) | 1/67 | (1%) |
|  | Medical management | 0/46 | (0%) | 1/21 | (5%) | 1/67 | (1%) |
| **DAPT regimen** | Aspirin and Clopidogrel | 27/46 | (59%) | 14/21 | (67%) | 41/67 | (61%) |
|  | Aspirin and Prasugrel | 2/46 | (4%) | 1/21 | (5%) | 3/67 | (4%) |
|  | Aspirin and Ticagrelor | 16/46 | (35%) | 6/21 | (29%) | 22/67 | (33%) |
|  | Clopidogrel | 1/46 | (2%) | 0/21 | (0%) | 1/67 | (1%) |
| **DAPT exposure time;^a^ n (%)** | ≤6 month | 18/43 | (42%) | 9/21 | (43%) | 27/64 | (42%) |
|  | >6 months | 25/43 | (58%) | 12/21 | (57%) | 37/64 | (58%) |
| **DAPT exposure time^a^ for ≤6 months (median; IQR months)** |  | 2.0 | (1.9, 3.0) | 1.3 | (1.0, 2.9) | 2.0 | (1.0, 3.0) |
| **DAPT exposure time^a^ for >6 months (median; IQR months)** |  | 12.0 | (12.0, 12.0) | 11.8 | (10.6, 12.7) | 12.0 | (12.0, 12.0) |

CABG – coronary artery bypass grafting; DAPT – dual antiplatelet therapy; IQR - interquartile range; PCI – percutaneous coronary intervention; PIL – participant information leaflet; SD – standard deviation

^a^Data missing from four participants who were sent a Participant Information Leaflet but did not attend.

**appendix D – Example participant study booklet**

The participant study booklet contains a demographics questionnaire followed by two baseline EQ-5D questionnaires (EQ-5D-3L and EQ-5D-5L) for assessing the participants own health. Some participants completed the EQ-5D-3L first and some completed the EQ-5D-5L first depending on the colour-coded study booklet randomly allocated to them at the beginning of the study. These questionnaires were completed before the focus group interviews commenced. On the subsequent pages, four more EQ-5D questionnaires were provided each associated with one of two vignettes describing an individual experiencing either a minor or major bleeding event while on antiplatelet therapy. Each EQ-5D questionnaire was prefaced with instructions on how the elicitation exercise should be completed followed by one of the two vignettes. Vignette A described an individual experiencing a minor bleed, whereas Vignette B described an individual experiencing a major bleed. At the bottom of each EQ-5D questionnaire there was a supplementary question that asked the participant how long they would expect their health-related quality of life to be affected by the bleeding event described in the respective vignette. Each participant completed both an EQ-5D-3L and EQ-5D-5L questionnaire for each of the two vignettes. The order of which was dependent on the colour-coded study booklet randomly allocated to them at the beginning of the study, in that some participants completed the EQ-5D for Vignette A first and others for Vignette B first. The four EQ-5D questionnaires associated with the two vignettes were completed after the completion of the focus group interviews. It should be noted that the EuroQol Research Foundation approved the use of the modified EQ-5D questionnaires on June 21, 2017 for the conduct of this study.

**Today’s date Study ID**

/

/

**About You**

1. Are you male or female? ***Please*** ***Tick ✓ One Box***

Male 🞏 Female 🞏

1. What is your date of birth?

/

/

1. What is the postcode of your main address?
2. Which of the following best describes your ethnic origin? ***Please*** ***Tick ✓ One Box***

| **White** |  | **Asian or Asian British** |  |
| --- | --- | --- | --- |
| British | 🞏 | Bangladeshi | 🞏 |
| Irish | 🞏 | Indian | 🞏 |
| Any other White background | 🞏 | Pakistani | 🞏 |
|  |  | Any other Asian background | 🞏 |
|  |  |  |  |
| **Mixed** |  | **Black or Black British** |  |
| White and Asian | 🞏 | African | 🞏 |
| White and Black African | 🞏 | Caribbean | 🞏 |
| White and Black Caribbean | 🞏 | Any other Black background | 🞏 |
|  |  |  |  |
| **Chinese** |  | Any **other** ethnic background  ***(Please say what in the box below)*** |  |
| Chinese | 🞏 |  | |

1. How many months have you been taking dual antiplatelet medication (aspirin plus clopidogrel; aspirin plus prasugrel; aspirin plus ticagrelor) for your heart?

months ***(Answer should be between 0 and 12 months)***

**By placing a tick in one box in each group below, please indicate which statements best describe your own health state today.**

**Mobility**

I have no problems in walking about 

I have some problems in walking about 

I am confined to bed 

**Self-Care**

I have no problems with self-care 

I have some problems washing or dressing myself 

I am unable to wash or dress myself 

**Usual Activities** *(e.g. work, study, housework, family or leisure activities)*

I have no problems with performing my usual activities 

I have some problems with performing my usual activities 

I am unable to perform my usual activities 

**Pain/Discomfort**

I have no pain or discomfort 

I have moderate pain or discomfort 

I have extreme pain or discomfort 

**Anxiety/Depression**

I am not anxious or depressed 

I am moderately anxious or depressed 

I am extremely anxious or depressed 

Under each heading, please tick the **ONE** box that best describes your health **TODAY**

**Mobility**

I have no problems in walking about 

I have slight problems in walking about 

I have moderate problems in walking about 

I have severe problems in walking about 

I am unable to walk about 

**Self-Care**

I have no problems washing or dressing myself 

I have slight problems washing or dressing myself 

I have moderate problems washing or dressing myself 

I have severe problems washing or dressing myself 

I am unable to wash or dress myself 

**Usual Activities** *(e.g. work, study, housework, family or leisure activities)*

I have no problems doing my usual activities 

I have slight problems doing my usual activities 

I have moderate problems doing my usual activities 

I have severe problems doing my usual activities 

I am unable to do my usual activities 

**Pain/Discomfort**

I have no pain or discomfort 

I have slight pain or discomfort 

I have moderate pain or discomfort 

I have severe pain or discomfort 

I have extreme pain or discomfort 

**Anxiety/Depression**

I am not anxious or depressed 

I am slightly anxious or depressed 

I am moderately anxious or depressed 

I am severely anxious or depressed 

I am extremely anxious or depressed 

**Please read the statement below. Try to imagine you are the patient and you have experienced the event described in the statement.**

**Now think about how this event may affect how you feel. On the next page please indicate which statements best describe how you would feel if you were the patient experiencing the event described in the statement below by placing a tick in one box for each of the five groups.**

*You are a patient currently receiving antiplatelet (‘blood-thinning’) treatment to reduce your risk of developing a blood clot and potentially experiencing another coronary event (‘heart attack’). This morning you experienced a bleeding event (e.g., bleeding from a cut or scrape, bleeding from the nose or mouth/gums or bruising). This bleeding event did NOT cause you to seek advice and/or treatment from a healthcare professional or visit the hospital. The bleeding event did, however, make you consider not taking your antiplatelet therapy at your next schedule dose.*

**Mobility**

I have no problems in walking about 

I have some problems in walking about 

I am confined to bed 

**Self-Care**

I have no problems with self-care 

I have some problems washing or dressing myself 

I am unable to wash or dress myself 

**Usual Activities** *(e.g. work, study, housework, family or leisure activities)*

I have no problems with performing my usual activities 

I have some problems with performing my usual activities 

I am unable to perform my usual activities 

**Pain/Discomfort**

I have no pain or discomfort 

I have moderate pain or discomfort 

I have extreme pain or discomfort 

**Anxiety/Depression**

I am not anxious or depressed 

I am moderately anxious or depressed 

I am extremely anxious or depressed 

**Supplementary question**

Based on the description of the bleeding event in the statement on the previous page and your responses to the five questions above how long would you expect your quality of life to be affected by the bleeding event? Please assume that the bleeding event described in the statement only occurs once. ____ Years ____ Months ____ Days

**Please read the statement below. Try to imagine you are the patient and you have experienced the event described in the statement.**

**Now think about how this event may affect how you feel. On the next page please indicate which statements best describe how you would feel if you were the patient experiencing the event described in the statement below by placing a tick in one box for each of the five groups.**

*You are a patient currently receiving antiplatelet (‘blood-thinning’) treatment to reduce your risk of developing a blood clot and potentially experiencing another coronary event (‘heart attack’). This morning you experienced a bleeding event (e.g., persistent nose bleed, blood in your bowel movement or bleeding from your bottom, vomiting blood or bleeding in your eye). The bleeding event causes you concern and therefore you decide to seek advice and/or treatment from a healthcare professional or visit the hospital.*

**Mobility**

I have no problems in walking about 

I have some problems in walking about 

I am confined to bed 

**Self-Care**

I have no problems with self-care 

I have some problems washing or dressing myself 

I am unable to wash or dress myself 

**Usual Activities** *(e.g. work, study, housework, family or leisure activities)*

I have no problems with performing my usual activities 

I have some problems with performing my usual activities 

I am unable to perform my usual activities 

**Pain/Discomfort**

I have no pain or discomfort 

I have moderate pain or discomfort 

I have extreme pain or discomfort 

**Anxiety/Depression**

I am not anxious or depressed 

I am moderately anxious or depressed 

I am extremely anxious or depressed 

**Supplementary question**

Based on the description of the bleeding event in the statement on the previous page and your responses to the five questions above how long would you expect your quality of life to be affected by the bleeding event? Please assume that the bleeding event described in the statement only occurs once. ____ Years ____ Months ____ Days

**Please read the statement below. Try to imagine you are the patient and you have experienced the event described in the statement.**

**Now think about how this event may affect how you feel. On the next page please indicate which statements best describe how you would feel if you were the patient experiencing the event described in the statement below by placing a tick in one box for each of the five groups.**

*You are a patient currently receiving antiplatelet (‘blood-thinning’) treatment to reduce your risk of developing a blood clot and potentially experiencing another coronary event (‘heart attack’). This morning you experienced a bleeding event (e.g., bleeding from a cut or scrape, bleeding from the nose or mouth/gums or bruising). This bleeding event did NOT cause you to seek advice and/or treatment from a healthcare professional or visit the hospital. The bleeding event did, however, make you consider not taking your antiplatelet therapy at your next schedule dose.*

**Mobility**

I have no problems in walking about 

I have slight problems in walking about 

I have moderate problems in walking about 

I have severe problems in walking about 

I am unable to walk about 

**Self-Care**

I have no problems washing or dressing myself 

I have slight problems washing or dressing myself 

I have moderate problems washing or dressing myself 

I have severe problems washing or dressing myself 

I am unable to wash or dress myself 

**Usual Activities** *(e.g. work, study, housework, family or leisure activities)*

I have no problems doing my usual activities 

I have slight problems doing my usual activities 

I have moderate problems doing my usual activities 

I have severe problems doing my usual activities 

I am unable to do my usual activities 

**Pain/Discomfort**

I have no pain or discomfort 

I have slight pain or discomfort 

I have moderate pain or discomfort 

I have severe pain or discomfort 

I have extreme pain or discomfort 

**Anxiety/Depression**

I am not anxious or depressed 

I am slightly anxious or depressed 

I am moderately anxious or depressed 

I am severely anxious or depressed 

I am extremely anxious or depressed 

**Supplementary question**

Based on the description of the bleeding event in the statement on the previous page and your responses to the five questions above how long would you expect your quality of life to be affected by the bleeding event? Please assume that the bleeding event described in the statement only occurs once. ____ Years ____ Months ____ Days

**Please read the statement below. Try to imagine you are the patient and you have experienced the event described in the statement.**

**Now think about how this event may affect how you feel. On the next page please indicate which statements best describe how you would feel if you were the patient experiencing the event described in the statement below by placing a tick in one box for each of the five groups.**

*You are a patient currently receiving antiplatelet (‘blood-thinning’) treatment to reduce your risk of developing a blood clot and potentially experiencing another coronary event (‘heart attack’). This morning you experienced a bleeding event (e.g., persistent nose bleed, blood in your bowel movement or bleeding from your bottom, vomiting blood or bleeding in your eye). The bleeding event causes you concern and therefore you decide to seek advice and/or treatment from a healthcare professional or visit the hospital.*

**Mobility**

I have no problems in walking about 

I have slight problems in walking about 

I have moderate problems in walking about 

I have severe problems in walking about 

I am unable to walk about 

**Self-Care**

I have no problems washing or dressing myself 

I have slight problems washing or dressing myself 

I have moderate problems washing or dressing myself 

I have severe problems washing or dressing myself 

I am unable to wash or dress myself 

**Usual Activities** *(e.g. work, study, housework, family or leisure activities)*

I have no problems doing my usual activities 

I have slight problems doing my usual activities 

I have moderate problems doing my usual activities 

I have severe problems doing my usual activities 

I am unable to do my usual activities 

**Pain/Discomfort**

I have no pain or discomfort 

I have slight pain or discomfort 

I have moderate pain or discomfort 

I have severe pain or discomfort 

I have extreme pain or discomfort 

**Anxiety/Depression**

I am not anxious or depressed 

I am slightly anxious or depressed 

I am moderately anxious or depressed 

I am severely anxious or depressed 

I am extremely anxious or depressed 

**Supplementary question**

Based on the description of the bleeding event in the statement on the previous page and your responses to the five questions above how long would you expect your quality of life to be affected by the bleeding event? Please assume that the bleeding event described in the statement only occurs once. ____ Years ____ Months ____ Days

**appendix E – Sources of utility decrements reported in decision analytic models**

A summary of the sources of utility decrements reported in decision analytic model for dual antiplatelet therapy is provided in the table below. Only one study [1] directly stated the source of/methods used to derive the reported decrements. Utility decrements were mainly derived based on assumptions,[2-4] unpublished data from trial sponsors [5] or listed as being obtained from a compendium of values;[6, 7] no utility decrements for bleeds were identified from these compedia. Three studies [8-10] cited multiple references as the source of the reported decrements and included one reference in common, namely a decision analytic model that used a utility decrement of -0.03 for bleeds that result in short-term morbidity.[11] This decrement was derived from a consensus of three medical internists who designated a health state utility value of 0.75 for one-month or a utility decrement of -0.0208 for short-term morbidity bleeds in elderly patients with atrial fibrillation.[12] Other sources cited, identified after retrieving multiple references used standard gamble methods to elicit utility values for major bleeds (0.841) from elderly patients with atrial fibrillation,[13] an assumption of a utility value of 0.8 for two days or utility decrement of ‑0.00110 for a minor haemorrhage in patients with chronic atrial fibrillation [14] or methods indiscernible based on an inaccessible report [15] and utility values for bleeds not reported in the cited reference.[16]

| **Author**  **[ref]** | **Source one and values reported** | **Source two and values reported** |
| --- | --- | --- |
| Greenhalgh  [2] | **Major bleed:**  UK population norms derived from Kind [17]; disutility for major bleed (25% decrement to UK population norms (free of disease) for 14 days; -0.007) based on assumption. | NA |
| Garg  [8] | **Minor bleed:**  Shah [18] reports utility value of 0.8 for two days (-0.00110), which were used in a model comparing various antithrombotic therapies in patients with AF.  **Extracranial major bleed:**  Two references are listed, but no clear synthesis methods are described as to how the information from each of the two references were used to obtain a final estimate.  Shah [18] reports utility value of 0.8 for one- month (-0.0167) which were used in a model comparing various antithrombotic therapies in patients with AF.  Augustovski [11] reports utility value of 0.97 for one-year period (-0.03) after event (bleeds that result in only short-term morbidity or noncerebral bleeding that required transfusion); equivalent to one-week deducted from overall survival. Was used in a model comparing aspirin or no aspirin for primary prevention of cardiovascular disease. | Shah [18] estimates derived from Thomson [13] who used standard gamble method to elicit utility values for major bleeding (0.841 SD 0.172) from elderly patients with AF and Fryback [16] who used the SF-36, Quality of Well-being index and time trade-off methods to obtain health state utility values for 28 conditions, none of which were bleeding events, from a random community-based sample of adults in the US. Unclear how these two sources were combined to obtain final estimates.  Augustovski [11] estimates derived from Naglie [12] who used consensus of three internists to determine the utility value (0.75 for one-month; -0.0208) for short-term morbidity bleeds in elderly patients with chronic nonvalvular AF receiving either warfarin, aspirin or no treatment. Not clear how Augustovski obtained a utility decrement of -0.03 from the information presented by Naglie. |
| Kazi  [9] | A number of references are listed under the general heading of bleeding, but no attempt has been made to assign specific reference to the different types of bleeding considered (minor, extracranial and CABG-related). In addition, no clear synthesis methods are described as to how the information from each of the references were used to obtain the final estimates.  Garg [8] reports utility decrement of -0.002 for minor bleeds, which were used in a model comparing different durations of DAPT in a ACS with PCI population.  Schleinitz [19] reports utility decrement of -0.005 for GI bleeding based on assumption, which were used in a model comparing clopidogrel and aspirin for secondary prevention in patients with a prior MI, stroke or PAD.  Freeman [20] reports utility value of 0.8 for 2 weeks (-0.00769) for major haemorrhage other than ICH and 0.8 for 2 days (-0.00110) for minor haemorrhage, which were used in a model comparing dabigatran and warfarin for patients receiving either dabigatran or warfarin for stroke prevention in AF.  Cohen [21] reports utility decrement of -1 quality adjusted week for short-term morbidity of vascular complications based on estimated duration of hospitalisation and recuperation of vascular complication event for patients with, single-vessel coronary disease treated by stenting or conventional angioplasty. Was used in a model comparing stenting and angioplasty in patients with symptomatic, single-vessel coronary disease. | Garg [8] estimates derived from Shah [18] and Augustovski [11]; see row 2 for more details.  Freeman [20] estimates for minor harmorrhage derived from O’Brien [14] who assumed a utility value of 0.8 for 2 days (-0.00110) for a minor haemorrhage, which was used in a model comparing ximelagatran, warfarin and aspirin in patients with chronic AF.  Freeman [20] estimates for major haemorrhage other than ICH derived from Thomson [13] who used standard gamble method to elicit utility values for major bleeding (0.841 SD 0.172) from elderly patients with AF and Fryback [16] who used the SF-36, Quality of Well-being index and time trade-off methods to obtain health state utility values for 28 conditions, none of which were bleeding events, from a random community-based sample of adults in the US. Unclear how these two sources were combined to obtain final estimates. |
| Liew  [5] | **Minor and major bleeds:**  Mean utility values were obtained from the study sponsors of the PLATO trial (comparison of ticagrelor and clopidogrel in ACS patients),[22] but no further details provided. | NA |
| Gupta  [3] | **GI haemorrhage:**  Cohen [21] reports utility decrement of -1 quality adjusted week for short-term morbidity of vascular complications based on estimated duration of hospitalisation and recuperation of vascular complication event for patients with, single-vessel coronary disease treated by stenting or conventional angioplasty. Was used in a model comparing stenting and angioplasty in patients with symptomatic, single-vessel coronary disease. | NA |
| Schleinitz  [1] | **GI bleed:**  Reported utility decrement (-0.005) based on assumption. |  |
| Latour-Perez  [4] | **Serious haemorrhage:**  Eckman [23] reports a utility value of 0.87 based on assumption relying on clinical experience for a bleeding event in patients with underlying heart disease receiving anticoagulant therapy. Was used in a model comparing anticoagulation therapy to no anticoagulation therapy in patients with heart disease. | NA |
| Jiang  [6] | **Nonfatal bleeding:**  Sullivan [24] reports utility decrements for a number of chronic conditions based on ICD-9 codes using the EQ-5D-3L in a US population; not clear where utility decrement for nonfatal bleeding was obtained as no such value is reported by Sullivan. | NA |
| Wang  [10] | **Major bleeding:**  Coleman [25] reports utility decrement for major bleeding of 0.02 for 1 year, which was used in a model comparing universal antiplatelet therapy to platelet reactivity assay driven antiplatelet therapy in patients with ACS. The estimate is supported by four references: Crespin;[26] Pignone;[27] Augustovski;[11] and Meenan,[28] but no clear synthesis methods are described as to how the information from each of the references were used to obtain a final estimate. | Crespin [26] reports a utility toll during the month of a GI bleed of 0.75 (-0.0208), which was used in a model comparing ticagrelor to genotype-deriven antiplatelet therapy for secondary prevention after ACS. The estimate is supported by three references: Pignone;[27] Augustovski;[11] and Meenan [28], but no clear synthesis methods are described as to how the information from each of the references were used to obtain a final estimate.  Pignone [27] reports a utility value of 0.94 for one-year (-0.06) for GI bleeding, which was used in a model comparing aspirin to no therapy for primary prevention of cardiovascular disease. The estimate is supported by one reference: Augustovski.[11]  Augustovski [11] estimates derived from Naglie [12] who used consensus of three internists to determine the utility value (0.75 for one-month; -0.0208) for short-term morbidity bleeds in elderly patients with chronic nonvalvular AF receiving either warfarin, aspirin or no treatment. Not clear how Augustovski obtained a utility decrement of -0.03 from the information presented by Naglie.  Meenan [28] reports a utility value of 0.997 for GI bleed, which was used in a model comparing echocardiography studies in newly diagnosed ischaemic stroke patients. This estimate is supported by one reference: Matchar [15] a Agency for Healthcare Research and Quality (AHRQ) report of a simulation model for studying the costs and outcomes of the natural history of stroke, but the report is not available online. |
| Jiang  [7] | **Nonfatal bleeding:**  Sullivan [24] reports utility decrements for a number of chronic conditions based on ICD-9 codes using the EQ-5D-3L in a US population; not clear where utility decrement for nonfatal bleeding was obtained as no such value is reported by Sullivan. | NA |

ACS – acute coronary syndrome; AF – atrial fibrillation; CABG – coronary artery bypass grafting; DAPT – dual antiplatelet therapy; GI – gastrointestinal; ICH – intracranial haemorrhage; MI – myocardial infarction; PAD - peripheral arterial disease; PCI – percutaneous coronary intervention

**appendix F – Quality assessment and relevance of utility decrements from the included studies**

The results of the quality and relevance assessment is provided in the table below. Only three studies [5, 29, 30] were judged to have patient characteristics very closely matched to our population of interest (i.e., post-coronary intervention on DAPT) and therefore of high relevance. The remaining studies used patients judged to be closely related (e.g., single-vessel disease treated with stenting or unstable angina on DAPT),[1, 3] or not to be closely related (e.g., general population, elderly atrial fibrillation or stroke patients and heart disease patients on anticoagulant therapy)[2, 4, 6-10] and therefore of moderate and low relevance respectively.

In terms of the quality/free from bias assessment, it was difficult to ascertain details concerning responses rates, loss to follow-up and missing data for the majority of the studies. Even for studies that did report details for one or more the characteristics,[2, 8, 9, 29, 30] reasons for deficiencies or how they were accounted for were not reported. There were additional difficulties assessing the risk of bias for three of the studies,[8-10] where multiple sources were used in estimating the utility decrements and no details provided concerning the synthesis methods used to combine the information. Three studies [1, 3, 4] obtained utility decrements for bleeds based on assumptions, which made the questions concerning response rates, loss to follow-up and missing data not applicable. Overall, the identified studies were judged to be at high risk of bias, given the lack of detailed reporting.

Most studies using a generic preference-based instrument provided adequate details of the version and tariff used, delivered the instrument as intended and applied it to its intended population.[2, 5-7, 29, 30] The remaining studies using valuation methods to elicit utility decrements (e.g., time-trade-off, standard gamble)[8, 9] or studies that based estimated utility decrements on assumptions/expert consensus [1, 3, 4, 10] provided very little detail to judge whether the approaches were appropriate.

Finally, none of the included studies were completely in line with the requirements for health-state utility values outlined in the NICE reference case.[31] The two studies that were the closest to the requirements were Greenhalgh et al.[2] who used EQ-5D-3L utility values aged matched from the UK general population and applied an assumed utility decrement from these values for a bleeding event and Amin et al.[30] who used responses to the EQ-5D-3L from post-PCI patients receiving DAPT who experienced either minor or major bleeds, but used the US EQ-5D-3L tariff to derive utility decrements.

|  | **Amin**  **[29]** | **Amin**  **[30]** | **Greenhalgh [2]** | **Garg**  **[8]** | **Kazi**  **[9]** | **Liew**  **[5]** | **Gupta**  **[3]** | **Schleinitz [1]** | **Latour Perez [4]** | **Jiang**  **[6]** | **Wang**  **[10]** | **Jiang**  **[7]** |
| --- | --- | --- | --- | --- | --- | --- | --- | --- | --- | --- | --- | --- |
| **Relevance to the decision problem** | | | | | | | | | | | | |
| How closely do the patient characteristics in the study match those described in the decision problem? | Very close; post-AMI on DAPT | Very close; post-PCI on DAPT | Not close; age matched UK general population | Not close; elderly AF patients and US adults | Not close; elderly AF patients and US adults | Very close; post-ACS on DAPT | Close; single-vessel disease patients treated by stenting | Close; unstable angina or non-Q-wave MI on DAPT | Not close; heart disease patients on anti-coagulant therapy | Not close; general population with self- reported medical diagnoses | Not close; elderly AF patients and stroke patients | Not close; general population with self- reported medical diagnoses |
| Does respondent selection and recruitment result in a population comparable to that being modelled? | Yes | Yes | Somewhat | No | No | Yes | NA; estimate based on assumption | NA; estimate based on assumption | NA; estimate based on assumption | Unclear | NA; estimate based on expert consensus and unknown source | Unclear |
| Do the inclusion / exclusion criteria exclude any individuals? | Yes; if transferred from another facility >25 hours after index event | Yes; those who died, missing baseline or 6-month data, incomplete or un-validated hospital records | Yes; individuals in institutions, hostels, elderly homes or bed and breakfast accommod-ation | Yes; history of bleeds, falls and excessive alcohol intake | Yes; history of bleeds, falls and excessive alcohol intake | Yes; excluded if needed oral anti-coagulation therapy or had risk of brady-cardia | NA; estimate based on assumption | NA; estimate based on assumption | NA; estimate based on assumption | Not reported | NA; estimate based on expert consensus and unknown source | Not reported |
| How closely do the inclusion criteria match people who would receive the intervention in routine practice? | Very close | Very close | Not close | Not close | Not close | Very close | NA; estimate based on assumption | NA; estimate based on assumption | NA; estimate based on assumption | Unclear | NA; estimate based on expert consensus and unknown source | Unclear |
| **Quality assessment – free from bias** | | | | | | | | | | | | |
| Is the precision of the estimate reflected in the variance around any estimate used in the model? | Yes | Yes | No | No | Yes; range noted for sensitivity analysis | No | No | Yes; range noted to create distribution | Yes; range noted for sensitivity analysis | Yes; range noted for sensitivity analysis | Yes; range noted for sensitivity analysis | Yes; range noted for sensitivity analysis |
| Are response rates reported and if so are the rates likely to be a threat to the validity of the estimated values? | No; potential threat to validity | No; potential threat to validity | Yes; 24% refused to take part; potential threat to validity | Yes; 23% of patients will not have AF diagnosis recorded in medical records; potential threat to validity; 11% refused particip-ation | Yes; 23% of patients will not have AF diagnosis recorded in medical records; potential threat to validity; 11% refused particip-ation | No; potential threat to validity | NA; estimate based on assumption | NA; estimate based on assumption | NA; estimate based on assumption | No; potential threat to validity | NA; estimate based on expert consensus and unknown source | No; potential threat to validity |
| How large is the loss to follow-up and are reasons given? | 16% with missing follow-up; no reasons provided | 16.5% with missing follow-up; no reasons provided | NA; no follow-up | NA; no follow-up | NA; no follow-up | Not reported | NA; estimate based on assumption | NA; estimate based on assumption | NA; estimate based on assumption | Not reported | NA; estimate based on expert consensus and unknown source | Not reported |
| Are any loss to follow-up reported likely to threaten the validity of the estimates? | Potential threat to validity | Potential threat to validity | NA; no follow-up | NA; no follow-up | NA; no follow-up | Not reported | NA; estimate based on assumption | NA; estimate based on assumption | NA; estimate based on assumption | Not reported | NA; estimate based on expert consensus and unknown source | Not reported |
| What are the levels of missing data and how are they dealt with? | Not reported | 25% missing data; excluded from study | Very small number of missing data points for each domain; Unclear how they were handled | Not reported | Not reported | Not reported | NA; estimate based on assumption | NA; estimate based on assumption | NA; estimate based on assumption | Not reported | NA; estimate based on expert consensus and unknown source | Not reported |
| Are there details on the causes of missing data? | No | Yes | No | Not reported | Not reported | Not reported | NA; estimate based on assumption | NA; estimate based on assumption | NA; estimate based on assumption | Not reported | NA; estimate based on expert consensus and unknown source | Not reported |
| Could any missing data reported threaten the validity of the estimates? | Potential threat to validity | Potential threat to validity | Unlikely threat to validity | Potential threat to validity | Potential threat to validity | Potential threat to validity | NA; estimate based on assumption | NA; estimate based on assumption | NA; estimate based on assumption | Potential threat to validity | NA; estimate based on expert consensus and unknown source | Potential threat to validity |
| **Utility values are measured and valued appropriately** | | | | | | | | | | | | |
| If valuation methods are used, are they used appropriately? | NA | NA | NA | Unclear, details not reported | Unclear, details not reported | NA | No; assumption based on average duration of hospitalisation | No; assumption no details | No; assumption based on clinical experience | NA | No; expert consensus | NA |
| Does the valuation method provide preference-based values anchored at 1 as equivalent to full health and 0 as equivalent to dead? | NA | NA | NA | Yes | Yes | NA | Unclear | Unclear | Unclear | NA | Unclear | NA |
| Are adequate details of the valuation method provided to allow judgement on appropriateness? | NA | NA | NA | No | No | NA | No | No | No | NA | No | NA |
| Are adequate details of the preference-based method provided? | Yes | Yes | Yes | NA | NA | Somewhat; no details of the tariff used with the EQ-5D | NA | NA | NA | Yes | NA | Yes |
| Was the generic preference-based measure delivered as intended? | Somewhat; only used EQ-5D VAS | Yes | Yes | NA | NA | Yes | NA | NA | NA | Yes | NA | Yes |
| Is the measure used for the group it was intended? | Yes | Yes | Yes | NA | NA | Yes | NA | NA | NA | Yes | NA | Yes |
| If a health state is valued using a vignette, can the appropriateness of the vignette be assessed? | NA | NA | NA | NA | NA | NA | NA | NA | NA | NA | NA | NA |
| **In line with reimbursement agency requirements (i.e., align with the National Institute for Health and Care Excellence (NICE) reference case)** | | | | | | | | | | | | |
| Is the geographical area of recruitment relevant for the reimbursement agency? | US; No | US; No | UK; Yes | UK and US; Somewhat | UK and US; Somewhat | US; No | US; No | US; No | US; No | US; No | US; No | US; No |
| Does the measure used to collect utility values match the requirements of the decision problem and reimbursement agency? | Somewhat; only used EQ-5D VAS | Yes;  EQ-5D-3L | Yes;  EQ-5D-3L | No; combin-ation of SG, Quality of Well-being index and TTO and expert opinion | No; combin-ation of SG, Quality of Well-being index and TTO, assumption and expert opinion | Yes;  EQ-5D-3L | No; assumption | No; assumption | No; assumption | Yes;  EQ-5D-3L | No; expert opinion and unknown source | Yes;  EQ-5D-3L |
| Who completes the measure and does it satisfy the requirements of the decision problem and reimbursement agency? | Patients; Yes | Patients; Yes | General population; No | Patients / clinical assumption; Somewhat | Patients / clinical assumption; Somewhat | Patients; Yes | Clinical assumption; No | Clinical assumption; No | Clinical assumption; No | General population / patients; Somewhat | Clinical assumption; No | General population / patients; Somewhat |
| Was mode of administration standardised across participants and in line with reimbursement agency requirements? | Yes | Unclear; via patient interview | Yes | Yes | Yes | Unclear; mode not specified | NA | NA | NA | Yes | NA | Yes |
| Who values the health states and does this satisfy the requirements of the reimbursement authority of interest? | Patients with VAS; No | General population; Yes | General population; Yes | Patients / clinical assumption; No | Patients / clinical assumption; No | Unclear; not reported | Clinical assumption; No | Clinical assumption; No | Clinical assumption; No | General population; Yes | Clinical assumption; No | General population; Yes |
| What techniques is used to value the health state and does this satisfy the requirements of the reimbursement authority of interest? | VAS; No | TTO, but US tariff; Somewhat | TTO, UK tariff; Yes | Combin-ation of SG, TTO and expert consensus; Somewhat | Combin-ation of SG, TTO, assumption and expert consensus; Somewhat | Unclear; not reported | Assumption based on the average duration of hospitalisation; No | Assumption with no details; No | Assumption based on clinical experience; No | TTO, but US tariff; Somewhat | Combin-ation of expert consensus and unknown source; Somewhat | TTO, but US tariff; Somewhat |

ACS – acute coronary syndrome; AF – atrial fibrillation; AMI – acute myocardial infarction; DAPT – dual antiplatelet therapy; MI – myocardial infarction; NA – not applicable; PCI – percutaneous coronary intervention; SG – standard gamble; TTO – time-trade-off; UK – United Kingdom; United States; VAS – visual analogue scale

**appendix G – Full regression results**

**Minor Bleed**

Dependent variable: United Kingdom EQ-5D-3L health state utility value

|  | **Coefficient** | **95% Confidence Interval** |
| --- | --- | --- |
| Bleeding event identifier | -0.120 | -0.252 to 0.0121 |
| Baseline health state utility value | 0.776 | 0.473 to 1.0800 |
| Age | -0.00284 | -0.00936 to 0.00369 |
| Sex (Male reference) |  |  |
| Female | 0.0327 | -0.306 to 0.372 |
| Intervention (PCI reference) |  |  |
| CABG | -0.0477 | -0.210 to 0.115 |
| Medical Management | 0.0153 | -0.342 to 0.373 |
| Days since started DAPT^a^ | 0.0000220 | -0.000455 to 0.000499 |
| Constant | 0.364 | -0.128 to 0.857 |

CABG – coronary artery bypass grafting; DAPT – dual antiplatelet therapy; PCI – percutaneous coronary intervention

^a^Days between the date of the focus group and the date the participant commenced DAPT therapy. The date the participant commenced DAPT therapy was derived from the screening questionnaire used during recruitment.

**Major Bleed**

Dependent variable: United Kingdom EQ-5D-3L health state utility value

|  | **Coefficient** | **95% Confidence Interval** |
| --- | --- | --- |
| Bleeding event identifier | -0.239 | -0.384 to -0.0933 |
| Baseline health state utility value | 0.541 | 0.206 to 0.876 |
| Age | 0.00403 | -0.00316 to 0.0112 |
| Sex (Male reference) |  |  |
| Female | -0.180 | -0.553 to 0.194 |
| Intervention (PCI reference) |  |  |
| CABG | -0.0741 | -0.253 to 0.105 |
| Medical Management | -0.0848 | -0.478 to 0.309 |
| Days since started DAPT^a^ | -0.000349 | -0.000875 to 0.000177 |
| Constant | 0.197 | -0.346 to 0.740 |

CABG – coronary artery bypass grafting; DAPT – dual antiplatelet therapy; PCI – percutaneous coronary intervention

^a^Days between the date of the focus group and the date the participant commenced DAPT therapy. The date the participant commenced DAPT therapy was derived from the screening questionnaire used during recruitment.

**Minor Bleed**

Dependent variable: United States EQ-5D-3L health state utility value

|  | **Coefficient** | **95% Confidence Interval** |
| --- | --- | --- |
| Bleeding event identifier | -0.0863 | -0.175 to 0.00203 |
| Baseline health state utility value | 0.762 | 0.456 to 1.0680 |
| Age | -0.00148 | -0.00583 to 0.00287 |
| Sex (Male reference) |  |  |
| Female | 0.0489 | -0.178 to 0.276 |
| Intervention (PCI reference) |  |  |
| CABG | -0.0414 | -0.150 to 0.0675 |
| Medical Management | 0.0132 | -0.225 to 0.000346 |
| Days since started DAPT^a^ | 0.0000276 | -0.000291 to 0.000346 |
| Constant | 0.294 | -0.0853 to 0.674 |

CABG – coronary artery bypass grafting; DAPT – dual antiplatelet therapy; PCI – percutaneous coronary intervention

^a^Days between the date of the focus group and the date the participant commenced DAPT therapy. The date the participant commenced DAPT therapy was derived from the screening questionnaire used during recruitment.

**Major Bleed**

Dependent variable: United States EQ-5D-3L health state utility value

|  | **Coefficient** | **95% Confidence Interval** |
| --- | --- | --- |
| Bleeding event identifier | -0.164 | -0.260 to -0.0672 |
| Baseline health state utility value | 0.536 | 0.202 to 0.869 |
| Age | 0.00308 | -0.00166 to 0.00782 |
| Sex (Male reference) |  |  |
| Female | -0.102 | -0.349 to 0.146 |
| Intervention (PCI reference) |  |  |
| CABG | -0.0532 | -0.172 to 0.0655 |
| Medical Management | -0.0461 | -0.306 to 0.214 |
| Days since started DAPT^a^ | -0.000234 | -0.000581 to 0.000114 |
| Constant | 0.252 | -0.162 to 0.666 |

CABG – coronary artery bypass grafting; DAPT – dual antiplatelet therapy; PCI – percutaneous coronary intervention

^a^Days between the date of the focus group and the date the participant commenced DAPT therapy. The date the participant commenced DAPT therapy was derived from the screening questionnaire used during recruitment.

**Minor Bleed**

Dependent variable: EQ-5D-5L to United Kingdom EQ-5D-3L health state utility value

|  | **Coefficient** | **95% Confidence Interval** |
| --- | --- | --- |
| Bleeding event identifier | -0.0514 | -0.129 to 0.0262 |
| Baseline health state utility value | 0.760 | 0.570 to 0.950 |
| Age | -0.000527 | -0.00456 to 0.00350 |
| Sex (Male reference) |  |  |
| Female | -0.0199 | -0.215 to 0.175 |
| Intervention (PCI reference) |  |  |
| CABG | -0.0168 | -0.115 to 0.0811 |
| Medical Management | -0.205 | -0.408 to -0.00156 |
| Days since started DAPT^a^ | -0.000109 | -0.000389 to 0.000171 |
| Constant | 0.259 | -0.0247 to 0.542 |

CABG – coronary artery bypass grafting; DAPT – dual antiplatelet therapy; PCI – percutaneous coronary intervention

^a^Days between the date of the focus group and the date the participant commenced DAPT therapy. The date the participant commenced DAPT therapy was derived from the screening questionnaire used during recruitment.

**Major Bleed**

Dependent variable: EQ-5D-5L to United Kingdom EQ-5D-3L health state utility value

|  | **Coefficient** | **95% Confidence Interval** |
| --- | --- | --- |
| Bleeding event identifier | -0.193 | -0.315 to -0.0713 |
| Baseline health state utility value | 0.508 | 0.204 to 0.812 |
| Age | 0.00111 | -0.00509 to 0.00732 |
| Sex (Male reference) |  |  |
| Female | 0.0217 | -0.288 to 0.331 |
| Intervention (PCI reference) |  |  |
| CABG | 0.000178 | -0.149 to 0.149 |
| Medical Management | -0.178 | -0.503 to 0.147 |
| Days since started DAPT^a^ | -0.000101 | -0.000546 to 0.000344 |
| Constant | 0.331 | -0.107 to 0.769 |

CABG – coronary artery bypass grafting; DAPT – dual antiplatelet therapy; PCI – percutaneous coronary intervention

^a^Days between the date of the focus group and the date the participant commenced DAPT therapy. The date the participant commenced DAPT therapy was derived from the screening questionnaire used during recruitment.

**Minor Bleed**

Dependent variable: EQ-5D-5L to United States EQ-5D-3L health state utility value

|  | **Coefficient** | **95% Confidence Interval** |
| --- | --- | --- |
| Bleeding event identifier | -0.0505 | -0.102 to 0.00105 |
| Baseline health state utility value | 0.758 | 0.578 to 0.938 |
| Age | -0.000313 | -0.00300 |
| Sex (Male reference) |  |  |
| Female | 0.00758 | -0.122 to 0.137 |
| Intervention (PCI reference) |  |  |
| CABG | -0.0135 | -0.0786 to 0.0515 |
| Medical Management | -0.115 | -0.249 to 0.0197 |
| Days since started DAPT^a^ | -0.0000605 | -0.000246 to 0.000125 |
| Constant | 0.242 | 0.0313 to 0.452 |

CABG – coronary artery bypass grafting; DAPT – dual antiplatelet therapy; PCI – percutaneous coronary intervention

^a^Days between the date of the focus group and the date the participant commenced DAPT therapy. The date the participant commenced DAPT therapy was derived from the screening questionnaire used during recruitment.

**Major Bleed**

Dependent variable: EQ-5D-5L to United States EQ-5D-3L health state utility value

|  | **Coefficient** | **95% Confidence Interval** |
| --- | --- | --- |
| Bleeding event identifier | -0.140 | -0.222 to -0.0584 |
| Baseline health state utility value | 0.501 | 0.211 to 0.791 |
| Age | 0.000839 | -0.00327 to 0.00495 |
| Sex (Male reference) |  |  |
| Female | 0.0216 | -0.186 to 0.229 |
| Intervention (PCI reference) |  |  |
| CABG | -0.00152 | -0.102 to 0.0985 |
| Medical Management | -0.113 | -0.329 to 0.104 |
| Days since started DAPT^a^ | -0.0000486 | -0.000346 to 0.000248 |
| Constant | 0.368 | 0.0357 to 0.700 |

CABG – coronary artery bypass grafting; DAPT – dual antiplatelet therapy; PCI – percutaneous coronary intervention

^a^Days between the date of the focus group and the date the participant commenced DAPT therapy. The date the participant commenced DAPT therapy was derived from the screening questionnaire used during recruitment.

**Minor Bleed**

Dependent variable: United Kingdom EQ-5D-5L health state utility value

|  | **Coefficient** | **95% Confidence Interval** |
| --- | --- | --- |
| Bleeding event identifier | -0.0312 | -0.0992 to 0.0369 |
| Baseline health state utility value | 0.708 | 0.516 to 0.901 |
| Age | -0.0000328 | -0.00356 to 0.00349 |
| Sex (Male reference) |  |  |
| Female | 0.0259 | -0.144 to 0.196 |
| Intervention (PCI reference) |  |  |
| CABG | -0.0364 | -0.124 to 0.0509 |
| Medical Management | -0.125 | -0.304 to 0.0531 |
| Days since started DAPT^a^ | -0.0000942 | -0.000336 to 0.000147 |
| Constant | 0.280 | 0.0234 to 0.536 |

CABG – coronary artery bypass grafting; DAPT – dual antiplatelet therapy; PCI – percutaneous coronary intervention

^a^Days between the date of the focus group and the date the participant commenced DAPT therapy. The date the participant commenced DAPT therapy was derived from the screening questionnaire used during recruitment.

**Major Bleed**

Dependent variable: United Kingdom EQ-5D-5L health state utility value

|  | **Coefficient** | **95% Confidence Interval** |
| --- | --- | --- |
| Bleeding event identifier | -0.166 | -0.278 to -0.0549 |
| Baseline health state utility value | 0.459 | 0.139 to 0.779 |
| Age | 0.00130 | -0.00435 to 0.00694 |
| Sex (Male reference) |  |  |
| Female | 0.0131 | -0.269 to 0.295 |
| Intervention (PCI reference) |  |  |
| CABG | 0.0128 | -0.126 to 0.151 |
| Medical Management | -0.159 | -0.456 to 0.138 |
| Days since started DAPT^a^ | -0.0000126 | -0.000413 to 0.000387 |
| Constant | 0.366 | -0.0489 to 0.780 |

CABG – coronary artery bypass grafting; DAPT – dual antiplatelet therapy; PCI – percutaneous coronary intervention

^a^Days between the date of the focus group and the date the participant commenced DAPT therapy. The date the participant commenced DAPT therapy was derived from the screening questionnaire used during recruitment.

# references - appendicies

1. Schleinitz MD, Heidenreich PA: **A cost-effectiveness analysis of combination antiplatelet therapy for high-risk acute coronary syndromes: clopidogrel plus aspirin versus aspirin alone.** *Ann Intern Med* 2005, **142:**251-259.

2. Greenhalgh J, Bagust A, Boland A, Dwan K, Beale S, Fleeman N, McEntee J, Dundar Y, Richardson M, Fisher M: **Prasugrel (Efient(R)) with percutaneous coronary intervention for treating acute coronary syndromes (review of TA182): systematic review and economic analysis.** *Health Technol Assess* 2015, **19:**1-130.

3. Gupta N, Nayak R, Grisolano SW, Buckles DC, Tadros PN: **Defining patients at high risk for gastrointestinal hemorrhage after drug-eluting stent placement: a cost utility analysis.** *J Interv Cardiol* 2010, **23:**179-187.

4. Latour-Perez J, Navarro-Ruiz A, Ridao-Lopez M, Cervera-Montes M: **Using clopidogrel in non-ST-segment elevation acute coronary syndrome patients: a cost-utility analysis in Spain.** *Value Health* 2004, **7:**52-60.

5. Liew D, De Abreu Lourenco R, Adena M, Chim L, Aylward P: **Cost-effectiveness of 12-month treatment with ticagrelor compared with clopidogrel in the management of acute coronary syndromes.** *Clin Ther* 2013, **35:**1110-1117 e1119.

6. Jiang M, You JH: **CYP2C19 LOF and GOF-Guided Antiplatelet Therapy in Patients with Acute Coronary Syndrome: A Cost-Effectiveness Analysis.** *Cardiovasc Drugs Ther* 2016.

7. Jiang M, You JH: **Cost-effectiveness analysis of personalized antiplatelet therapy in patients with acute coronary syndrome.** *Pharmacogenomics* 2016, **17:**701-713.

8. Garg P, Galper BZ, Cohen DJ, Yeh RW, Mauri L: **Balancing the risks of bleeding and stent thrombosis: a decision analytic model to compare durations of dual antiplatelet therapy after drug-eluting stents.** *Am Heart J* 2015, **169:**222-233 e225.

9. Kazi DS, Garber AM, Shah RU, Dudley RA, Mell MW, Rhee C, Moshkevich S, Boothroyd DB, Owens DK, Hlatky MA: **Cost-effectiveness of genotype-guided and dual antiplatelet therapies in acute coronary syndrome.** *Ann Intern Med* 2014, **160:**221-232.

10. Wang Y, Yan BP, Liew D, Lee VW: **Cost-effectiveness of cytochrome P450 2C19 *2 genotype-guided selection of clopidogrel or ticagrelor in Chinese patients with acute coronary syndrome.** *Pharmacogenomics J* 2017.

11. Augustovski FA, Cantor SB, Thach CT, Spann SJ: **Aspirin for primary prevention of cardiovascular events.** *Journal of General Internal Medicine* 1998, **13:**824-835.

12. Naglie IG, Detsky AS: **Treatment of chronic nonvalvular atrial fibrillation in the elderly: a decision analysis.** *Med Decis Making* 1992, **12:**239-249.

13. Thomson R, Parkin D, Eccles M, Sudlow M, Robinson A: **Decision analysis and guidelines for anticoagulant therapy to prevent stroke in patients with atrial fibrillation.** *Lancet* 2000, **355:**956-962.

14. O'Brien CL, Gage BF: **Costs and effectiveness of ximelagatran for stroke prophylaxis in chronic atrial fibrillation.** *JAMA* 2005, **293:**699-706.

15. Matcher DB, Samsa GP: *Secondary and Tertiary Prevention of Stroke. Patient Outcomes Research Team (PORT) Final Report - Phase 1.* Rockville, MD: Agency for Healthcare Research and Quality; 2000.

16. Fryback DG, Dasbach EJ, Klein R, Klein BE, Dorn N, Peterson K, Martin PA: **The Beaver Dam Health Outcomes Study: initial catalog of health-state quality factors.** *Med Decis Making* 1993, **13:**89-102.

17. Kind P, Hardman G, Macran S: *UK Population Norms for EQ-5D - Centre for Health Economics Discussion Paper (172).* York: University of York; 1999.

18. Shah SV, Gage BF: **Cost-effectiveness of dabigatran for stroke prophylaxis in atrial fibrillation.** *Circulation* 2011, **123:**2562-2570.

19. Schleinitz MD, Weiss JP, Owens DK: **Clopidogrel versus aspirin for secondary prophylaxis of vascular events: a cost-effectiveness analysis.** *Am J Med* 2004, **116:**797-806.

20. Freeman JV, Zhu RP, Owens DK, Garber AM, Hutton DW, Go AS, Wang PJ, Turakhia MP: **Cost-effectiveness of dabigatran compared with warfarin for stroke prevention in atrial fibrillation.** *Ann Intern Med* 2011, **154:**1-11.

21. Cohen DJ, Breall JA, Ho KK, Kuntz RE, Goldman L, Baim DS, Weinstein MC: **Evaluating the potential cost-effectiveness of stenting as a treatment for symptomatic single-vessel coronary disease. Use of a decision-analytic model.** *Circulation* 1994, **89:**1859-1874.

22. Wallentin L, Becker RC, Budaj A, Cannon CP, Emanuelsson H, Held C, Horrow J, Husted S, James S, Katus H, et al: **Ticagrelor versus clopidogrel in patients with acute coronary syndromes.** *N Engl J Med* 2009, **361:**1045-1057.

23. Eckman MH, Levine HJ, Salem DN, Pauker SG: **Making decisions about antithrombotic therapy in heart disease: decision analytic and cost-effectiveness issues.** *CHEST Journal* 1998, **114:**699S-714S.

24. Sullivan PW, Ghushchyan V: **Preference-Based EQ-5D Index Scores for Chronic Conditions in the United States.** *Medical decision making : an international journal of the Society for Medical Decision Making* 2006, **26:**410-420.

25. Coleman CI, Limone BL: **Cost-effectiveness of universal and platelet reactivity assay-driven antiplatelet therapy in acute coronary syndrome.** *Am J Cardiol* 2013, **112:**355-362.

26. Crespin DJ, Federspiel JJ, Biddle AK, Jonas DE, Rossi JS: **Ticagrelor versus genotype-driven antiplatelet therapy for secondary prevention after acute coronary syndrome: a cost-effectiveness analysis.** *Value Health* 2011, **14:**483-491.

27. Pignone M, Earnshaw S, Pletcher MJ, Tice JA: **Aspirin for the primary prevention of cardiovascular disease in women: a cost-utility analysis.** *Arch Intern Med* 2007, **167:**290-295.

28. Meenan RT, Saha S, Chou R, Swarztrauber K, Pyle Krages K, O'Keeffe-Rosetti MC, McDonagh M, Chan BK, Hornbrook MC, Helfand M: **Cost-effectiveness of echocardiography to identify intracardiac thrombus among patients with first stroke or transient ischemic attack.** *Med Decis Making* 2007, **27:**161-177.

29. Amin AP, Bachuwar A, Reid KJ, Chhatriwalla AK, Salisbury AC, Yeh RW, Kosiborod M, Wang TY, Alexander KP, Gosch K, et al: **Nuisance bleeding with prolonged dual antiplatelet therapy after acute myocardial infarction and its impact on health status.** *J Am Coll Cardiol* 2013, **61:**2130-2138.

30. Amin AP, Wang TY, McCoy L, Bach RG, Effron MB, Peterson ED, Cohen DJ: **Impact of Bleeding on Quality of Life in Patients on DAPT: Insights From TRANSLATE-ACS.** *J Am Coll Cardiol* 2016, **67:**59-65.

31. National Institute for Health and Care Excellence (NICE): *Guide to the methods of technology appraisal* London: NICE; April 2013.
